# The impact of natural climate variability on the global distribution of *Aedes aegypti*: a mathematical modelling study

**DOI:** 10.1016/S2542-5196(24)00238-9

**Published:** 2024-12-01

**Authors:** Alexander R Kaye, Uri Obolski, Lantao Sun, William S Hart, James W Hurrell, Michael J Tildesley, Robin N Thompson

**Affiliations:** Mathematics Institute University of Warwick, Coventry, UK; Zeeman Institute for Systems Biology and Infectious Disease Epidemiology Research University of Warwick, Coventry, UK; Department of Epidemiology and Preventive Medicine, School of Public Health, Faculty of Medicine, Tel Aviv University, Tel Aviv-Yafo, Israel; Porter School of the Environment and Earth Sciences, Faculty of Exact Sciences, Tel Aviv University, Tel Aviv-Yafo, Israel; Department of Atmospheric Science, Colorado State University, Fort Collins, CO, USA; Mathematical Institute, University of Oxford, Oxford, UK; Department of Atmospheric Science, Colorado State University, Fort Collins, CO, USA; Mathematics Institute, University of Warwick, Coventry, UK; Zeeman Institute for Systems Biology and Infectious Disease Epidemiology Research, University of Warwick, Coventry, UK; School of Life Sciences, University of Warwick, Coventry, UK; Mathematical Institute, University of Oxford, Oxford, UK

## Abstract

**Background:**

*Aedes aegypti* spread pathogens affecting humans, including dengue, Zika, and yellow fever viruses. Anthropogenic climate change is altering the spatial distribution of *Ae aegypti* and therefore the locations at risk of vector-borne disease. In addition to climate change, natural climate variability, resulting from internal atmospheric processes and interactions between climate system components (eg, atmosphere–land and atmosphere–ocean interactions), determines climate outcomes. However, the role of natural climate variability in modifying the effects of anthropogenic climate change on future environmental suitability for *Ae aegypti* has not been assessed fully. In this study, we aim to assess uncertainty arising from natural climate variability in projections of *Ae aegypti* suitability up to the year 2100.

**Methods:**

In this mathematical modelling study, we developed an ecological model in which *Ae aegypti* population dynamics depend on climate variables (temperature and rainfall). We used 100 projections of future climate from the Community Earth System Model, a comprehensive climate model that simulates natural climate variability as well as anthropogenic climate change, in combination with our ecological model to generate a range of equally plausible scenarios describing the global distribution of suitable conditions for *Ae aegypti* up to 2100. Each of these scenarios corresponds to a single climate projection, allowing us to explore the difference in *Ae aegypti* suitability between the most-suitable and the least-suitable projections.

**Findings:**

Our key finding was that natural climate variability generates substantial variation in future projections of environmental suitability for *Ae aegypti*. Even for projections generated under the same Shared Socioeconomic Pathway (SSP) scenario (SSP3–7.0), in 2100 climatic conditions in London might be suitable for *Ae aegypti* for 0–5 months of the year, depending on natural climate variability.

**Interpretation:**

Natural climate variability affects environmental suitability for important disease vectors. Some regions could experience vector-borne disease outbreaks earlier than expected under climate change alone.

**Funding:**

Engineering and Physical Sciences Research Council and Wellcome Trust.

## Introduction

Climate-sensitive infectious diseases pose a substantial threat to public health.^1^ Anticipating the locations in which future outbreaks are most likely to occur allows scarce surveillance resources to be deployed effectively.

Vector-borne diseases, and their vectors such as *Aedes aegypti*, are particularly sensitive to climate variations.^2^ Climate variables such as temperature and rainfall affect vector ecology, including vector lifespan,^3^ probability of egg survival,^4^ and development time from eggs to adults.^5^ Laboratory-based research has been complemented by observational capture-release studies^6,7^ and modelling analyses,^8–11^ demonstrating that the impacts of climate on vector dynamics are substantial and complex. Projecting the effects of future climate on vector-borne disease outbreaks therefore requires an understanding of the relationship between the climate and the ecology of vectors such as *Ae aegypti*.

Mathematical models have been developed and applied to study the impact of anthropogenic climate change on the spatial distribution of mosquitoes. For example, Kraemer and colleagues^12^ developed a machine-learning model to predict the current spatial distributions of both *Ae aegypti* and *Aedes albopictus* and then used it to project future changes in those distributions accounting for climate change, urbanisation, and increased connectivity between locations.^13^ Mordecai and colleagues^14^ and Ryan and colleagues^9^ considered models in which the basic reproduction number (*R*_0_) of *Aedes*-borne viruses is assumed to depend on temperature, and projected the future temperature suitability of locations in the Americas and globally for pathogen transmission. Parham and Michael^15^ developed a similar model for malaria in which *R*_0_ depends on temperature and rainfall, but did not use it to make global predictions. These and other similar studies have provided valuable insights into the effects of climate change on vector ecology and the potential for disease outbreaks. Such studies often consider different future socioeconomic pathways and variability in projections driven by different climate models.^13,16^ However, these represent only two of the potential sources of uncertainty in projections of future climate. A further source of uncertainty is natural climate variability, which will also strongly influence Earth’s future climate trajectory.^17^

Natural (or internal) climate variability^17–19^ refers to fluctuations in climate that occur even if there are no changes in the radiative (external) forcing of the planet. Natural climate variability results from internal processes within the climate system, such as interactions between the atmosphere, oceans, and land surface, and includes both predictable features (eg, the El Niño Southern Oscillation) and irreducible uncertainty because the climate system is chaotic. One approach for quantifying uncertainty due to natural climate variability is to create an ensemble of climate projections using the same climate model under a specified radiative forcing scenario, perturbing the initial conditions of each projection. By initiating the projections from slightly different states in this way, the chaotic nature of the system causes the climate trajectories to diverge. Since the different projections each contain year-on-year variability, but are out of phase, the forced anthropogenic climate change signal can be obtained by averaging over the climate projections (appendix p 10). The construction of ensembles of climate projections made up of large numbers of runs (typically 50–100 projections) differing only in perturbations to the initial conditions is a recent advance. Consideration of natural climate variability in this way, capturing a range of possible outcomes under a single socioeconomic development and greenhouse-gas emissions scenario, is of substantial interest in Earth system science. Despite the importance of natural climate variability in projections of future climate, a thorough investigation into the combined effects of anthropogenic climate change and natural climate variability on environmental suitability for disease vectors has not previously been undertaken.

Here, we address this gap and develop a mathematical modelling framework to demonstrate that natural climate variability affects projections of the global spatial distribution of suitable climate conditions for *Ae aegypti*. Our primary objective is to explore the extent to which uncertainty in the future of the climate based on chaotic processes affects the number of months that will be suitable for *Ae aegypti* in any given future year. In our analyses, we use a large ensemble of forward projections of temperature and rainfall in locations globally from the Community Earth System Model^20–22^ (CESM), each run under the same Shared Socioeconomic Pathway (SSP) scenario.^23^ The climate conditions at any future time can be thought of as a random sample from the CESM projections at that time.

We use the CESM projections as inputs to a model of climate suitability for *Ae aegypti*. We investigate the regions that might be expected to experience an expansion in suitable conditions for *Ae aegypti* over the remainder of the 21st century. We explore the impact of natural climate variability by assessing the variation in the number of months that are suitable for *Ae aegypti* each year between different climate projections. Our main goal is not to develop a new climate-sensitive ecological model, but instead to demonstrate the general principle that natural climate variability markedly affects projections of climate suitability for important disease vectors. We therefore not only generate projections using our model but we also consider projections based on a range of previously published models of environmental suitability for *Ae aegypti*. This research represents the first detailed study of the combined effects of climate change and natural climate variability on the temporal and global spatial distribution of environmental suitability for *Ae aegypti*, with implications for future vector-borne disease outbreak risks.

## Methods

### Future climate projections

For our main analyses, simulated data describing future climate states were obtained from the CESM (version 2)^21^ Large Ensemble Community Project (LENS2).^22^ The climate model dataset consisted of 100 equally plausible climate simulations run from 1850 to 2100, created by specifying only very small differences in the initial states between simulations.^22^ As previously mentioned, the projected effect of the externally forced anthropogenic climate change signal can be obtained by averaging out the noise of natural climate variability. In climate science, this is typically achieved by examining the ensemble mean of the 100 different climate simulations. Real-world climate dynamics are less smooth than this ensemble mean (appendix p 10), however, which illustrates the need to consider natural climate variability in addition to anthropogenic climate change when inferring the effects of future climate on environmental suitability for *Ae aegypti*.

The CESM projections used in our main analyses were generated under the assumption that socioeconomic changes up to the year 2100 occur under the SSP3–7.0 scenario. This specific scenario characterises a global future with fragmented efforts towards environmental sustainability and, therefore, moderate to high challenges in reaching the mitigation levels that are required to address climate change.^23^ The CESM LENS2 dataset provides the values of projected climate variables across a global grid consisting of 192 latitude and 288 longitude values. We considered the average air temperature (at a reference height of 2 m above the surface of the Earth) and total rainfall each month in each location for each CESM simulation individually in the period from 2020 to 2100. Since the parameters of the ecological model depend on daily rainfall rather than monthly rainfall, we then converted the average monthly rainfall to its corresponding average daily value.

### Ecological model

**Figure 1:**
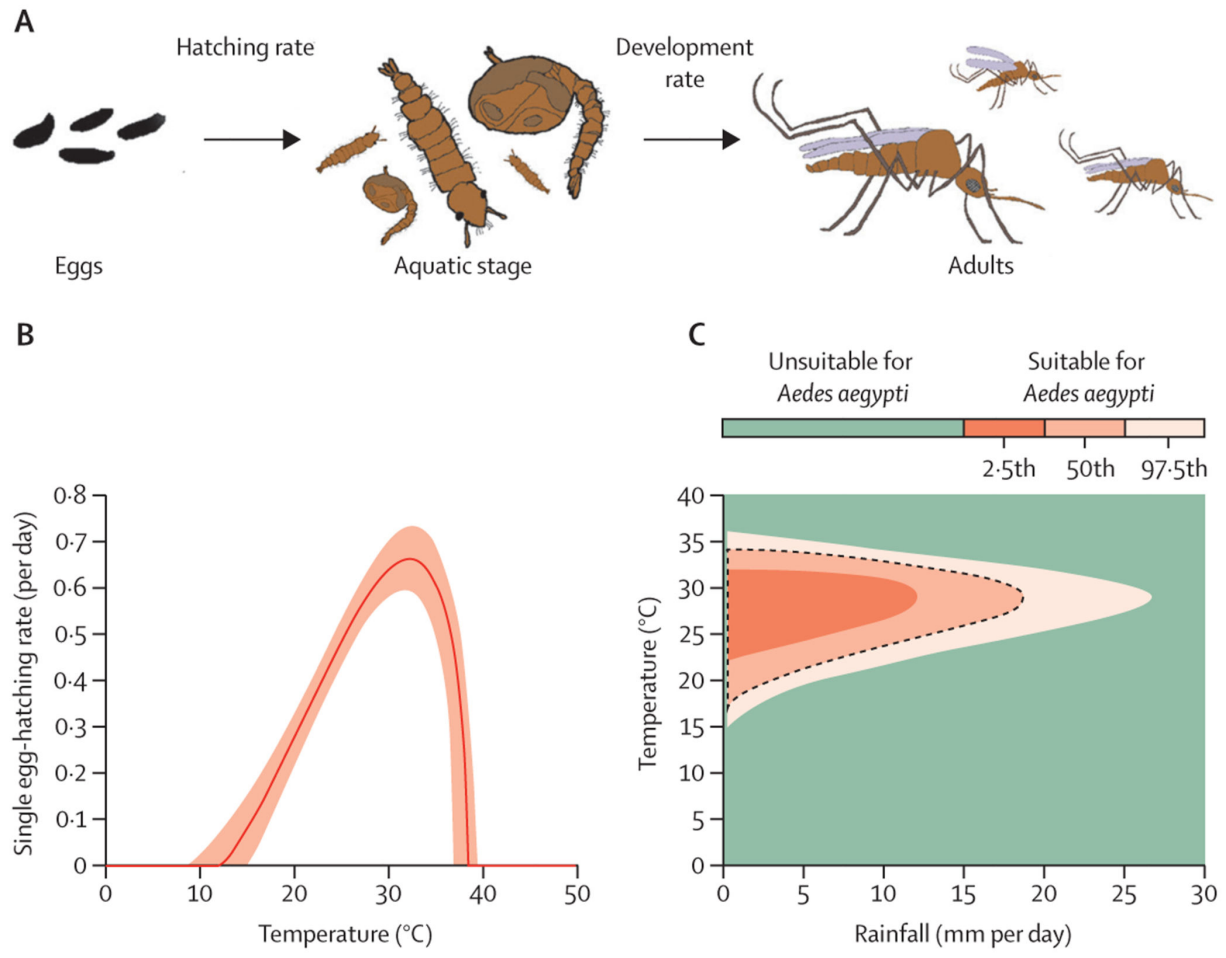
Climate-sensitive model of *Aedes aegypti* population dynamics (A) Lifecycle of *Ae aegypti*; each stage is represented by a different compartment in the ecological model. (B)Ecological parameters were assumed to depend on either temperature or rainfall. The dependence of the *Ae aegypti* egg-hatching rate on temperature is shown, estimated using data from a previous study^14^ (orange, median estimate; shaded area, 95% credible interval). (C) The ecological niche, describing temperature–rainfall values at which an *Ae aegypti* population can be sustained (orange), as derived from the ecological model. Uncertainty in the ecological niche was represented by different shades of orange (representing the 2·5th, 50th, and 97·5th percentile estimates) and arose because of uncertainty in the parameter estimates of the ecological model. The black dotted line indicates the outline of the median ecological niche.

In most of our analyses, *Ae aegypti* dynamics in each location were assumed to be represented by a compartmental, ordinary differential equation model in which members of the *Ae aegypti* population are divided according to their lifecycle stage, comprising eggs, aquatic larvae or pupae, and adults (figure 1A). Each model parameter, including the speed at which vectors move between these stages, was assumed to depend on either temperature or rainfall.

To infer the relationship between temperature and each temperature-dependent model parameter, we adapted the modelling framework of Mordecai and colleagues.^14^ In brief, a general functional form of the relationship between temperature and each model parameter was assumed. We fitted the precise temperature-dependent response to data from that study using Markov chain Monte Carlo (MCMC). As an example, the dependence of the egg-hatching rate (the rate at which eggs hatch and enter the aquatic stage) on temperature is shown in figure 1B. Since we adopted a Bayesian approach, we obtained a range of plausible temperature-dependent responses for each parameter (corresponding to different steps of the MCMC chain). The median egg-hatching rate at each temperature value was shown (orange in figure 1B, with the shaded area representing the 95% credible interval [CrI]). Further details about the ecological model and model fitting are given in the appendix (pp 1–8). Results from the model fitting procedure are also shown in the appendix (pp 11–14), along with responses analogous to the fitted curve in figure 1B, but for all temperature-dependent model parameters (p 15).

Two model parameters were assumed to depend on rainfall. The aquatic stage carrying capacity was assumed to increase with higher rainfall. By contrast, survival of aquatic-stage individuals decreases with higher rainfall, as larvae can be washed away. Relationships between rainfall and these model parameters were derived from first principles using a mechanistic approach (appendix pp 6–8).

Rather than simulating *Ae aegypti* population dynamics, we considered climate suitability for *Ae aegypti* by computing the ecological niche (combinations of temperature and rainfall at which the ecological model permits *Ae aegypti* survival). Specifically, for each set of ecological model parameter values (corresponding to different steps of the MCMC fitting procedure) individually, we calculated the equilibria of the ecological model at each possible temperature–rainfall combination. We then calculated the median ecological niche by identifying the temperature–rainfall values for which an equilibrium vector population size greater than zero was obtained in 50% of the parameter sets (with similar calculations done for the 2·5th and 97·5th percentile niches; figure 1C). We then used the median ecological niche to explore the number of months each year that are suitable for *Ae aegypti* in each of the climate projections from the CESM.

### Role of the funding source

The funders of this study had no role in data collection, data analysis, data interpretation, writing of the manuscript, or decision to submit this manuscript for publication.

## Results

**Figure 2:**
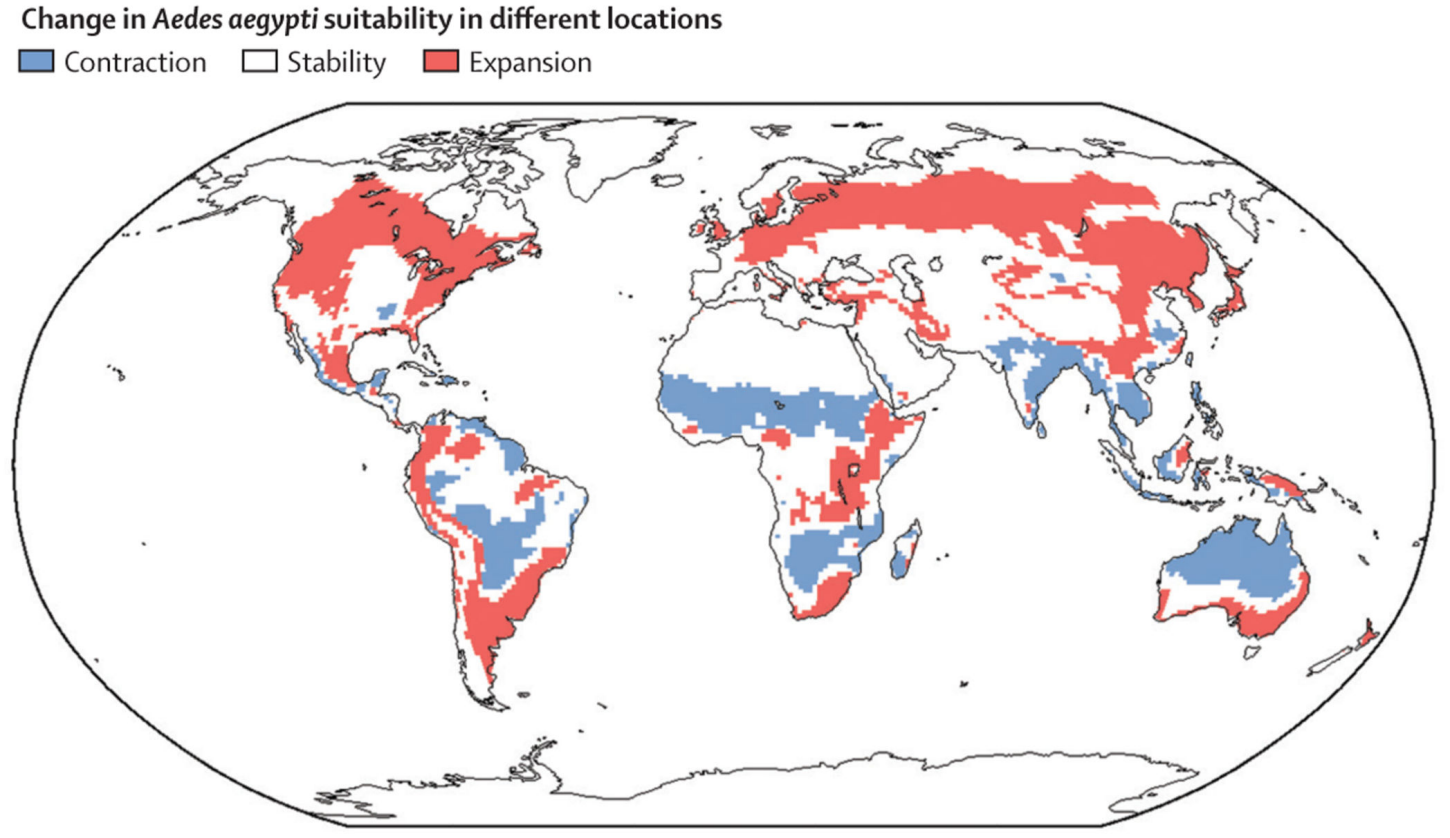
Locations that are expected to see an increase or decrease in the suitability of climatic conditions for *Aedes aegypti* Locations in which the number of months that are suitable for *Ae aegypti* increases by at least 1 month in 2100 compared with 2020 are shown in red. Locations with a corresponding decrease are shown in blue. These results were obtained by first calculating the change in the number of suitable months for each CESM projection individually, and then averaging the number of suitable months across all projections. We also did a supplementary analysis in which we averaged the climate simulations before calculating the number of suitable months for *Ae aegypti* in 2020 and 2100 (appendix p 20). CESM=Community Earth System Model.

**Figure 3:**
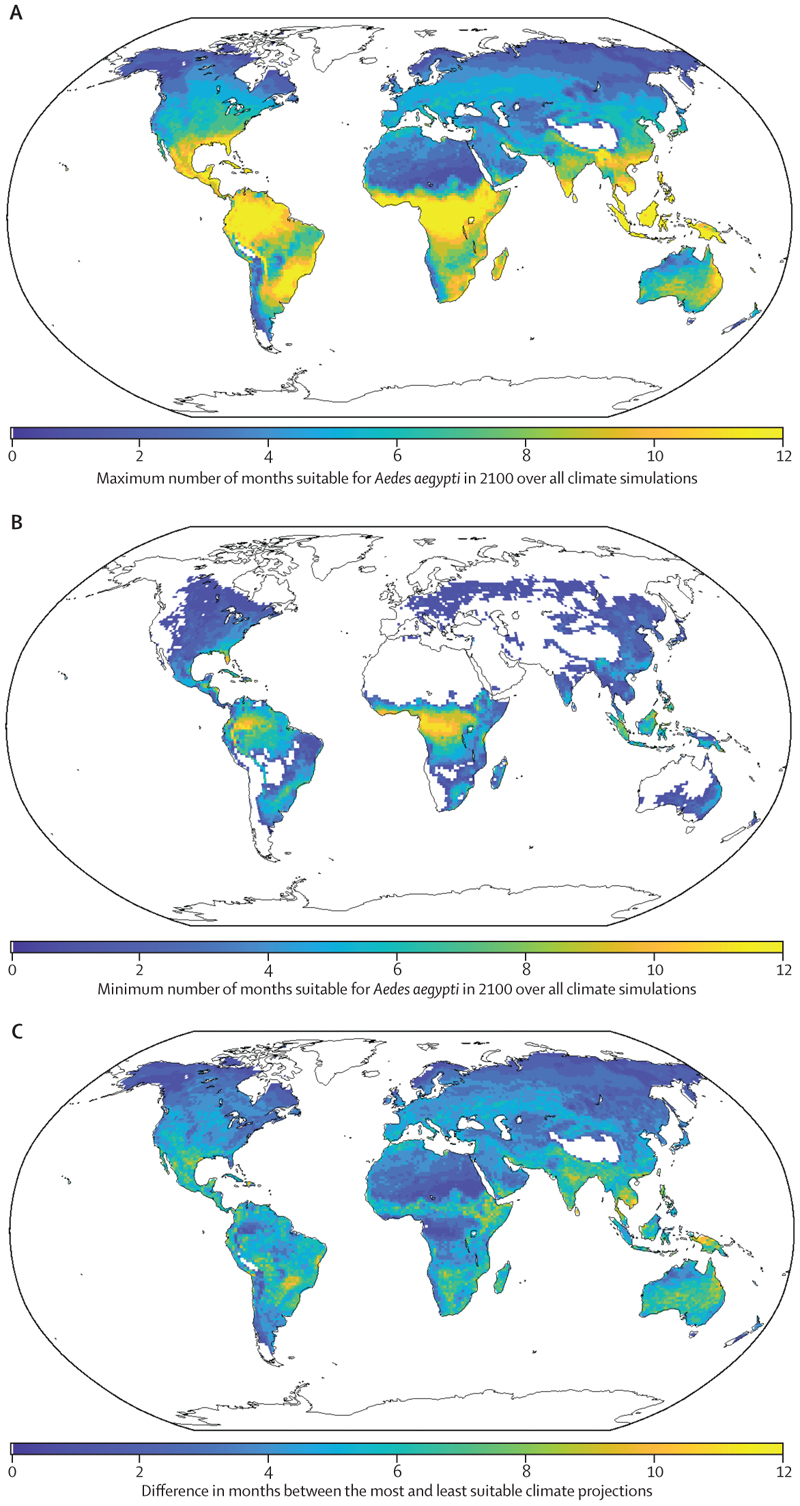
Impact of natural climate variability on future suitability for *Aedes aegypti* in different locations under SSP3–7.0 (A) Maximum number of months that are projected to be suitable for *Ae aegypti* in the year 2100. (B) Minimum number of months that are projected to be suitable for *Ae aegypti* in the year 2100. (C) Difference between the maximum and minimum number of months that are projected to be suitable for *Ae aegypti* in the year 2100. For each latitude–longitude value, the CESM projection corresponding to the most (A) or fewest (B) number of months that are suitable for *Ae aegypti* in the year 2100 was chosen. CESM=Community Earth System Model. SSP=shared socioeconomic pathway.

To understand the effect of temperature and rainfall on environmental suitability for *Ae aegypti*, we first computed the ecological niche (figure 1C). Uncertainty in the dependence of model parameter values on climate variables (eg, shaded region, figure 1B) led to uncertainty in the boundary of the ecological niche (different shades of orange, figure 1C). In our main analyses using this ecological model (figures 2, 3), we assumed that the ecological niche was characterised by its median estimate (black dotted line, figure 1C). We also validated the derived ecological niche using data from countries with observed *Ae aegypti* populations and locations of previous dengue outbreaks (appendix p 16).

For each CESM simulation and location, we calculated the number of months per year in which climate conditions were projected to be suitable for *Ae aegypti* survival according to the ecological niche. To check that our model generated results that were consistent with real-world *Ae aegypti* observations, we compared the predicted number of months that are suitable for *Ae aegypti* in different geographical locations globally in 2020 (averaging the number of months of suitability in each individual CESM simulation) against locations in which *Ae aegypti* populations have previously been observed (appendix p 17). We then computed mean change (across CESM simulations) in the number of months that were projected to be suitable for *Ae aegypti* survival between 2020 and 2100. We identified the locations that were expected to have suitable conditions for *Ae aegypti* for more months of the year in 2100 than 2020 (red, figure 2). Similarly, we identified the locations that were expected to have suitable conditions for *Ae aegypti* for fewer months of the year in 2100 than 2020 (blue, figure 2). Averaging over the suitability projections, as found in previous studies,^9^ under the assumed ecological model a poleward expansion of suitable environmental conditions for *Ae aegypti* is projected to occur during the 21st century. This finding was robust to the exact shape of the ecological niche. We also considered different assumptions about the relationship between rainfall and the rate at which aquatic-stage individuals are washed away, thereby generating different ecological niches, and consequently different suitability projections for *Ae aegypti* in 2100. A similar poleward spread is observed under these assumptions (appendix pp 18–19).

However, future *Ae aegypti* dynamics depend on natural climate variability, as characterised by the wide range of climate outcomes in the CESM simulations. In each location, we therefore considered the variability in the number of months each year in which climate conditions are projected to be suitable for *Ae aegypti* survival between CESM projections. The maximum number of months (across CESM projections) that are projected to be suitable for *Ae aegypti* survival in each location in the year 2100 is shown (figure 3A), with the equivalent minimum number of months in each location also depicted (figure 3B). Although the suitability projections (figure 3A, 3B) are not representative of any individual CESM projection, they demonstrate the wide variation in projected *Ae aegypti* suitability in each location between different CESM projections (figure 3C; appendix p 21).

**Figure 4:**
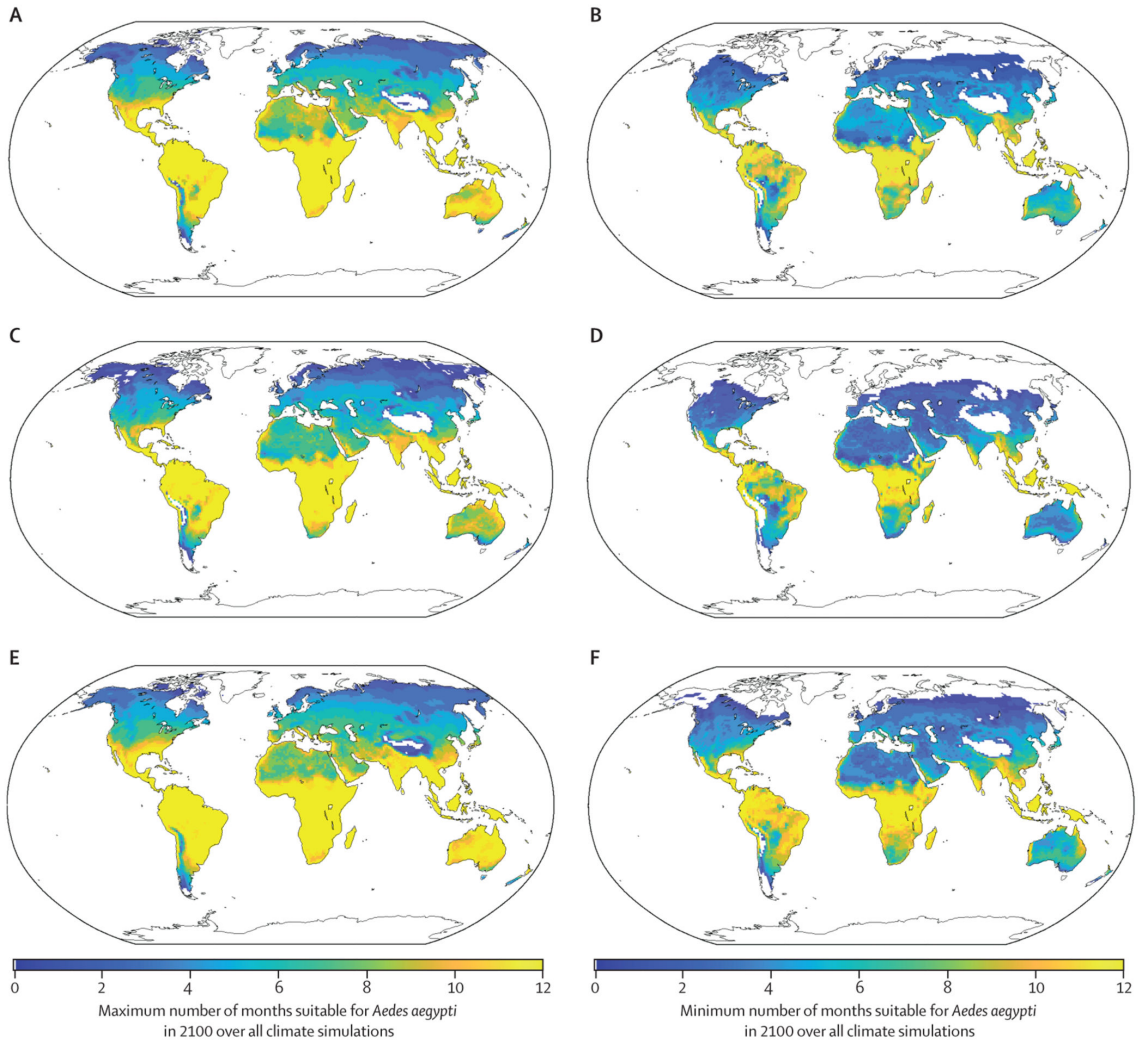
Impact of natural climate variability on future suitability for *Aedes aegypti* in different locations using ecological niches from the literature under SSP3–7.0 (A) Maximum number of months that are projected to be suitable for *Ae aegypti* in the year 2100 using the ecological niche from Mordecai and colleagues.^14^ (B) Minimum number of months that are projected to be suitable for *Ae aegypti* in the year 2100 using the ecological niche from Mordecai and colleagues.^14^ (C) Maximum number of months that are projected to be suitable for *Ae aegypti* in the year 2100 using the ecological niche from Ryan and colleagues.^9^ (D) Minimum number of months that are projected to be suitable for *Ae aegypti* in the year 2100 using the ecological niche from Ryan and colleagues.^9^ (E) Maximum number of months that are projected to be suitable for *Ae aegypti* in the year 2100 using the ecological niche from the model of Liu-Helmersson and colleagues.^16^ For this model, we derived the ecological niche by calculating the equilibria for different temperature–rainfall values under the baseline model parameterisation in that article (as in our main analyses, the ecological niche is assumed to represent temperature–rainfall values for which a positive equilibrium vector population size exists). (F) Minimum number of months that are projected to be suitable for *Ae aegypti* in the year 2100 using the ecological niche from the model of Liu-Helmersson and colleagues.^16^ SSP=shared socioeconomic pathway.

This finding highlights the importance of natural climate variability, particularly in geographical locations in which there is a substantial difference between the maximum and minimum numbers of months of suitability (figure 3A–C). As an example, in London, UK, where *Ae aegypti* do not currently inhabit, the number of months suitable for *Ae aegypti* survival in 2100 could be between 0 months (figure 3B) and 5 months (figure 3A), depending on natural climate variability. By contrast, if anthropogenic climate change alone is considered (ie, the mean of the CESM simulations is used to infer future climate suitability for *Ae aegypti*), then our model projects that 4 months will be suitable for *Ae aegypti* in the year 2100. Similarly, in Cape Town, South Africa, the number of months suitable for *Ae aegypti* survival in 2100 could be between 0 months (figure 3B) and 4 months (figure 3A), compared with 2 months when natural climate variability is averaged out (ie, using 100-member ensemble mean values for the climate variables). In Islamabad, Pakistan, the number of months suitable for *Ae aegypti* survival in 2100 is projected to be between 2 months (figure 3B) and 7 months (figure 3A), compared with 5 months on the basis of the ensemble mean. A particularly large amount of variability in future environmental suitability for *Ae aegypti* between CESM projections can be seen in some locations, including parts of Brazil, India, and Indonesia (figure 3C).

Although the analysis using our derived ecological niche (figure 3) allowed us to demonstrate that natural climate variability affects projections of environmental suitability for *Ae aegypti*, we conducted further analyses to demonstrate the robustness of this key finding to our modelling assumptions.

First, we repeated our analysis using ecological niches obtained from different models of climate-sensitive vector dynamics in the literature. We showed analogous results to the analysis using our derived ecological niche (figure 3) for ecological niches for *Ae aegypti* obtained from studies by Mordecai and colleagues,^14^ Ryan and colleagues,^9^ and Liu-Helmersson and colleagues (figure 4).^16^ We also considered ecological niches for *Aedes albopictus* (appendix p 22) and other disease vectors (appendix p 9). In each case that we examined, we found that natural climate variability drives substantial uncertainty in future projections of environmental suitability for important disease vectors, except in some scenarios in which climate conditions will not be suitable for vector survival at all or will be suitable all year round.

Although our main analyses used climate projections based on SSP3–7.0 because of the availability of 100 CESM simulations under that SSP, a smaller number of CESM simulations (19 in total) are available on the basis of the more optimistic SSP2–4.5. To demonstrate the robustness of our conclusion that natural climate variability affects projections of environmental suitability for *Ae aegypti* to the assumed SSP, we repeated the analysis using alternative CESM simulations (appendix p 23). We again found a substantial difference between the minimum and maximum number of months that are projected to be suitable for *Ae aegypti* globally in 2100.

Although climate projections up to 2100 allowed us to consider changes in environmental suitability for *Ae aegypti* over a long timescale, our finding that natural climate variability affects projections of suitability for disease vectors also holds over shorter timescales (eg, we consider the analogous results to those shown in figure 3A, B using projected temperature and rainfall in the year 2060 in the appendix [p 24]).

## Discussion

Vector-borne pathogens, such as the dengue, Zika, and yellow-fever viruses, are responsible for more than 700 000 deaths each year^24^ and have particularly devastating effects on populations in low-income and middle-income countries. Understanding the future threat to planetary public health posed by these pathogens requires changes in environmental suitability for their vectors to be projected.

In this study, we constructed a climate-sensitive ecological modelling framework that describes how environmental suitability for *Ae aegypti* populations changes as climate variables (temperature and rainfall) vary. We used this model to derive an ecological niche determining the conditions under which self-sustaining *Ae aegypti* populations are possible (figure 1C). Using climate projections from the CESM covering the period from 2020 to 2100, we then projected the locations that will be suitable for *Ae aegypti* during the 21st century and inferred the locations in which suitability for *Ae aegypti* is expected to change (figure 2). Similarly to previous modelling studies,^9^ we found that a poleward spread of suitable conditions for *Ae aegypti* is expected. However, the key difference between our study and previous analyses is that, owing to using a large ensemble of 100 equally plausible climate projections from the CESM, we were able to do a thorough investigation into the impact of natural climate variability on projections of the global spatial distribution of environmental suitability for *Ae aegypti*. Natural climate variability is well studied by climate scientists and is as important as anthropogenic climate change in shaping Earth’s future climate trajectory, especially regionally and even over many decades.^17,25^ One previous study has considered natural climate variability in the context of future *Ae aegypti* dynamics,^26^ but only a small number of climate-model projections were available for use in that research, meaning that the full effect of natural climate variability on future environmental suitability for *Ae aegypti* had not been properly assessed.

We found that natural climate variability has a substantial effect on the suitability of different locations to harbour *Ae aegypti* in the future (figure 3). When estimates are constructed accounting for anthropogenic climate change alone (ie, using the ensemble mean of simulations from one or more climate models), the wide variation in possible future climatic conditions in different locations due to natural climate variability is ignored. This is important, particularly as natural climate variability might sometimes generate suitable climate conditions for *Ae aegypti* in locations that are projected to be unsuitable for *Ae aegypti* when natural climate variability is not accounted for. Thus, outbreaks of vector-borne disease might occur in some places earlier than expected under anthropogenic climate change alone. We demonstrated the robustness of our conclusion that natural climate variability affects future environmental suitability for disease vectors to the ecological niche defining suitable conditions for vectors (figure 4; appendix p 9), the vector species (appendix p 9, 22), and the assumed SSP underlying the climate projections (appendix p 23).

Our finding that natural climate variability could lead to some years with higher-than-expected suitability for disease vectors highlights the importance of surveillance for both vectors and vector-borne pathogens. In attempting to prevent outbreaks, interventions that reduce vector prevalence such as removal of standing water and use of larvicides are crucial, coupled with public information campaigns to promote good practice. Some locations that do not currently harbour *Aedes* populations might be expected to see initial seasonal outbreaks of *Aedes*-borne pathogens that are relatively small, as has been the case (for example) in countries in southern Europe. Although this type of outbreak is probable, the potential for natural climate variability to lead to some years with high levels of environmental suitability for vectors could exacerbate the effect of outbreaks. As such, it is essential for policy advisors in countries that do not currently experience outbreaks of *Aedes*-borne pathogens to consider natural climate variability when assessing policy-relevant questions, for example how soon will it be until local outbreaks of those diseases occur? And when outbreaks happen, how large will they be? A related question for epidemiological modellers is how uncertainty in modelling projections, driven by phenomena such as natural climate variability, can be communicated to policy advisors effectively. Policy advisors should consider how to account for uncertainty in future projections when formulating policy decisions.

In our approach, variability between climate projections arose because of small perturbations in the initial conditions. As such, we were able to characterise the impact of variability due to the chaotic nature of the climate system. We used the projections to determine the uncertainty in the number of months that will be suitable for *Ae aegypti* in specific years in future. We note that year-on-year changes in suitability for vectors, as reflected in individual CESM simulations, may also have a role in the extent to which vector populations can persist. To explore year-on-year changes in environmental suitability, we did a supplementary analysis in which we considered the variation in the number of months that are projected to be suitable for *Ae aegypti* both between years in the period 2090–2100 and between CESM simulations (appendix p 25). Considering London, UK, Cape Town, South Africa, and Islamabad, Pakistan as specific examples, we found that, on average, environmental conditions will be suitable for *Ae aegypti* every year in that period. However, there are climate projections in which some years in that period are not suitable for *Ae aegypti* at all (ie, there were simulations that led to zero months of environmental suitability for *Ae aegypti* in some years), with potential implications for the ability of the vector to persist over several years. This finding again highlights the importance of considering natural climate variability when assessing future environmental suitability for disease vectors.

Like any quantitative analysis, our modelling study involved assumptions and simplifications. Our main goal was to investigate the extent to which natural climate variability affects projections of future suitability for disease vectors rather than to make precise quantitative predictions. Consequently, additional details should be included in the model underlying most of our analyses if it is used to guide vector or pathogen surveillance programmes. For example, the presence of *Ae aegypti* does not only depend on climate suitability, but also on a range of other factors including human behaviour. Future iterations of our model of suitability for *Ae aegypti* could account for changing human population sizes and geographical heterogeneity in socioeconomic development.^16,27^ Expansion of cities, including urbanisation in Africa, would be expected to affect future environmental suitability for *Ae aegypti*. Another factor that could be considered is the method of water storage in different locations, given that water storage containers act as *Ae aegypti* breeding sites.^28^ Although many previous models of *Ae aegypti* suitability have included temperature as the only climate variable, we also chose to incorporate rainfall as a model input. The rationale for this choice was that rainfall has previously been found to modulate the presence of disease vectors,^13,16,29^ and indeed we found that projections from our model would be substantially different if spatial variation in rainfall was not accounted for (appendix p 26). We note that other climate variables, such as humidity, also affect *Ae aegypti* suitability, and further consideration could be given to how temperature and rainfall are included in our modelling framework (eg, finer resolution temporal variations could be considered). In addition, local microclimates and differences in temperature (eg, variations in temperatures between indoors and outdoors, and the presence of urban heat islands) would affect vector population dynamics, and therefore are worthy of further study. Although several extensions to our modelling framework are possible, we contend that inclusion of additional climate information would be unlikely to alter our key finding that natural climate variability affects projections of environmental suitability for disease vectors.

Although our model projections indicate that *Ae aegypti* are likely to spread to new locations, we also note that climate conditions in some places are projected to become less suitable for *Ae aegypti* during the 21st century (figure 2). We urge against interpreting this result to mean that the situation will become better in those locations. First, if climate conditions become unsuitable for *Ae aegypti*, for example because of temperatures becoming too hot, then a range of other problems may arise, including increased drought, storm severity, food insecurity, and population displacement. Second, our model did not include the possibility that the vectors adapt to allow survival in these adverse climate conditions,^30^ which might be particularly relevant over the long timescale that we are considering. If future studies explore the effect of vector adaptation on environmental suitability for vectors, it could be necessary to consider the effect of natural climate variability. Year-on-year climate variability has the potential to disrupt the ability of vectors to adapt, given that such variability adds noise to the climate change signal.

Despite the simplifying assumptions made in our modelling framework, the novelty in this research is the demonstration that the future spatial distribution of suitable environmental conditions for vectors of globally important pathogens depends not only on anthropogenic climate change but also on natural climate variability. It is therefore important that future projections of climate-sensitive ecological and epidemiological systems consider natural climate variability, alongside other types of climate uncertainty such as climate model uncertainty and scenario uncertainty. During the 21st century, vectors and the pathogens that they transmit will undoubtedly spread to new locations because of climate shifts. Careful climate monitoring and rigorous surveillance for vectors and vector-borne pathogens is essential. Surveillance strategies should be informed by the wide range of possible future climate scenarios, accounting for all potential sources of variability. This will allow more resilient public health infrastructure to be built than considering a single possible climate future alone. This is of clear importance for planetary public health.

## Supplementary Material

Supplementary appendix

## Data Availability

All data generated or analysed during this study, including computing code for reproducing our results, are available at www.github.com/KayeARK/Climate_Change_NCV_Ae_Aegypti.

## Research in context

### Evidence before this study

We searched PubMed for articles published in English from database inception to July 11, 2023, with the search terms “climate change”, “*Aedes aegypti*”, and “climate variability”. This returned four articles, all of which involved using mathematical modelling to investigate the impact of climate on vector-borne disease. However, one of these studies did not explicitly model climate variability at all and two only considered within-year seasonal weather variations. The other article explored the impact of interannual differences in climatic conditions on dengue incidence, but at a localised scale (for a single country). Expanding our search by only using “climate change” and “*Aedes aegypti*” as search terms, we found 151 studies, of which 15 made global projections of the impact of future climate on the spatial distribution of *Ae aegypti* and the pathogens they transmit. These studies identified climate and anthropogenic factors as important drivers of *Ae aegypti* presence or absence, including temperature, rainfall, humidity, urbanicity, and socioeconomic development. One study considered several climate scenarios from the same model but did not use a large ensemble of climate projections. None of these studies conducted a thorough analysis of the impact of natural climate variability on environmental suitability for *Ae aegypti* or other disease vectors.

### Added value of this study

We combined a climate-sensitive model of environmental suitability for *Ae aegypti* with forward projections (until 2100) from the Community Earth System Model (CESM). A full exploration of the effects of natural climate variability on environmental suitability for vectors requires a large ensemble of climate projections as generated by the CESM. Our study shows that natural climate variability, in addition to anthropogenic climate change, generates substantial uncertainty in the global spatial distribution of suitable conditions for *Ae aegypti* going forwards.

### Implications of all the available evidence

The risk of vector-borne disease outbreaks will continue to be exacerbated by climate change. Vectors will spread into regions in which they are currently absent, increasing the risk of outbreaks. Given that natural climate variability will affect which locations are most at risk, continual monitoring of climate variability is essential. Furthermore, rigorous surveillance for vectors and the pathogens they spread is crucial for global public health. Vector and pathogen surveillance planning, done as part of routine epidemic and pandemic preparedness, should be guided by a range of possible future climate scenarios that account for natural climate variability.
