# Supplementary material for: The impact of natural climate variability on the global distribution of Aedes aegypti: a mathematical modelling study
Source: Lancet Planet Health. Author manuscript; Available in PMC 2025 Jul 8. (PMC7617884; doi:10.1016/S2542-5196(24)00238-9)
Supplement: Supplementary appendix [file EMS206530-supplement-Supplementary_appendix.pdf]

THE LANCET
Planetary Health

Supplementary appendix

This appendix formed part of the original submission and has been peer reviewed. We post it as supplied by the authors.

Supplement to: Kaye AR, Obolski U, Sun L, et al. The impact of natural climate variability on the global distribution of *Aedes aegypti*: a mathematical modelling study. *Lancet Planet Health* 2024; **8:** e1079–87.

Supplementary Material for "The impact of natural climate variability on the global distribution of *Aedes aegypti*: a mathematical modelling study"

AR Kaye, U Obolski, L Sun, WS Hart, JW Hurrell, MJ Tildesley, RN Thompson

Supplementary Text

Supplementary details about the ecological model and its parameterisation

The ecological model

As described in the main text, in the ecological model the *Ae. aegypti* population is divided according to their life cycle stage: eggs (E), aquatic stage (larvae or pupae; A) and adult female mosquitoes (M). The compartmental, ordinary differential equation model is given by

$$\frac{dE}{dt} = a(T)M - b(T)E,$$

$$\frac{dA}{dt} = b(T)E\left(1 - \frac{A}{K(R)}\right) - c(R)A - d(T)A - f(T)A,$$

$$\frac{dM}{dt} = \frac{1}{2}f(T)A - g(T)M,$$

in which T indicates dependence on temperature (°C) and R indicates dependence on rainfall (mm day^{-1}).

The parameter $a(T)$ is the birth rate (eggs per adult female per day), $b(T)$ is the egg-to-aquatic development rate (in a setting in which the birth rate is not resource limited), $d(T)$ is the aquatic stage death rate, $f(T)$ is the aquatic-to-adult development rate and $g(T)$ is the adult death rate. Rainfall-dependence is included through the aquatic stage carrying capacity ($K(R)$) and the rate at which aquatic stage individuals are washed away ($c(R)$). The factor of

$\frac{1}{2}$ is included in the final equation as we are predominantly interested in female adults; male adults do not spread pathogens. However, we include male *Ae. aegypti* in the model up to the adult stage, since they contribute to competition for resources.

For fixed temperature and rainfall values, the non-zero equilibrium value of M under this model is given by

$$M^* = K(R)\left(\frac{f(T)}{2g(T)} - \frac{c(R) + d(T) + f(T)}{a(T)}\right).$$

The value of M^* is positive, so that a self-sustaining *Ae. aegypti* population is possible, when

$$\frac{f(T)}{2g(T)} > \frac{c(R) + d(T) + f(T)}{a(T)}.$$

This expression is used as the basis for the ecological niche shown in Fig 1C in the main text.

Temperature-dependent model parameters

The relationships between temperature and each temperature-dependent model parameter are determined based on the data reported by Mordecai *et al.*[1] Details for each model parameter are given below (further information about the functional forms of the fitted relationships are provided in the following subsection):

- Birth rate, $a(T)$: We fitted a Brière equation directly to data describing the number of eggs laid per adult female per day at different temperature values.
- Egg-to-aquatic development rate, $b(T)$: Data describing the egg-to-aquatic development rate were not available from the Mordecai *et al.*[1] study. We therefore estimated $b(T)$ indirectly by first fitting a Brière equation to data describing the egg-to-adult development rate as a function of temperature ($\gamma(T)$, say). Neglecting resource limitation, and the possibility that aquatic stage individuals

die or are washed away, the egg-to-adult development rate depends on both the egg-to-aquatic and aquatic-to-adult development rates. A previous study by Silva *et al.*[2] in which climate dependence was not considered found that the mean egg-to-aquatic and aquatic-to-adult development rates were 0.24 day^{-1} and 0.125 day^{-1}, respectively. Assuming that the ratio between these quantities applies at all temperatures, then $b(T) = \frac{0.125+0.24}{0.125}\gamma(T) = \frac{73}{25}\gamma(T)$.

- Aquatic-to-adult development rate, $f(T)$: Analogously to estimating the egg-to-aquatic development rate, $f(T) = \frac{73}{48}\gamma(T)$.
- Aquatic stage death rate, $d(T)$. Data were available[1] describing the probability that an individual survives from egg to adult at different temperatures ($p(T)$, say). We fitted a quadratic equation to these data. Neglecting resource limitation, and the possibility that aquatic stage individuals are washed away, gives $p(T) = \frac{f(T)}{d(T)+f(T)}$ and so $d(T) = f(T)\left(\frac{1}{p(T)} - 1\right)$.
- Adult death rate, $g(T)$. A quadratic equation was fitted directly to data describing the adult lifespan ($1/g(T)$) at different temperatures.

Parameters fits are shown for $a(T)$ (Fig S2), $\gamma(T)$ (Fig S3), $p(T)$ (Fig S4) and $1/g(T)$ (Fig S5), from which the posteriors for the temperature-dependent parameters of the ecological model were obtained (Fig S6A-D).

<u>Details of the temperature-dependent parameter fitting</u>

As described above, a Brière equation or a quadratic equation was fitted to determine the relationship between individual *Ae. aegypti* model parameters and temperature. Both of these functions have a similar peaked shape, but Brière equations are asymmetric whereas quadratic equations are symmetric about the peak value. We therefore chose whether to fit a Brière or quadratic equation to determine the relationship between temperature and each

fitted parameter according to whether the relevant data appeared to be symmetric about their peak. This approach was also used by Mordecai *et al.*[1]

The functional form of the fitted Brière equation for the *Ae. aegypti* birth rate is

$$a(T) = \max\Big(0, \mathrm{Re}\big(\alpha T(T - T_0)\sqrt{T_m - T}\big) + \mathcal{N}(0, \sigma^2)\Big), \qquad \text{(S1)}$$

in which $T_m > T_0$, $\mathrm{Re}(z)$ denotes the real part of the complex number z, and $\mathcal{N}(0, \sigma^2)$ represents Gaussian noise with mean zero and variance σ^2. An analogous equation was used when we characterised $\gamma(T)$ using a Brière equation. Similarly, the quadratic functional form for $p(T)$ is

$$p(T) = \max\big(0, -\alpha(T - T_0)(T - T_m) + \mathcal{N}(0, \sigma^2)\big), \qquad \text{(S2)}$$

in which $T_m > T_0$, with a similar equation for $1/g(T)$.

Here, we describe the details of the MCMC approach used to determine the sub-parameters α, T_0, T_m and σ^2 of $a(T)$. A similar method was used for all fitted temperature-dependent parameters (and we reused the same notation for the sub-parameters – i.e., α, T_0, T_m and σ^2 – in each case).

The Metropolis-Hastings algorithm was used. In each step of the MCMC chain, new values of α, T_0, T_m and σ^2 were proposed, each sampled from independent Gaussian proposal distributions with mean equal to the current value and variances 5×10^{-3}, 1, 0.1 and 1, respectively. These variance values (referred to as Σ values in Tables S1 and S2) were chosen to achieve an acceptance rate of around 0.234.[3] The Σ values for other fitted temperature-dependent parameters are shown in Tables S1 and S2, along with the priors used for each sub-parameter. The algorithm was run for 100,000 steps, including a burn-in of 50,000 steps.

We repeated the fitting procedure a further four times, each time starting the MCMC chain from a different initial state, allowing us to confirm convergence of the original chain using the Gelman-Rubin statistic (see captions to Figs S2-S5). When fitting the temperature-dependent response for $a(T)$, the initial value of α was sampled for each chain from a $U(1 \times 10^{-3}, 1 \times 10^{-1})$ distribution, the initial value of T_0 was sampled from a $U(0,20)$ distribution, the initial value of T_m was sampled from a $U(20,40)$ distribution and the initial value of σ^2 was sampled from a $U(1,10)$ distribution. The distributions from which the initial values were sampled for the other temperature-dependent responses are shown in Tables S1 and S2.

Table S1. Technical details of the MCMC procedure used to determine the temperature-dependent responses of $\boldsymbol{a(T)}$ and $\boldsymbol{\gamma(T)}$. Due to the asymmetric nature of the data for these parameters, Brière equations were used (equation S1). The notation $\Gamma(x, y)$ represents a gamma distribution with shape parameter x and scale parameter y, and $\mathrm{U}(x, y)$ is a continuous uniform distribution with bounds x and y. The initial value in the MCMC chain of each sub-parameter was sampled from the distributions listed in the fourth column.

Parameter	Sub-parameter	Prior	Initial value	Σ Value
Birth rate, $a(T)$	α	$\Gamma(2, 10^{-2})$	$\mathrm{U}(10^{-3}, 10^{-1})$	5×10^{-3}
	T_0	$\Gamma(10, 2)$	$\mathrm{U}(0, 20)$	1
	T_m	$\Gamma(10, 4)$	$\mathrm{U}(20, 40)$	0.1
	σ^2	$\mathrm{U}(0, 10)$	$\mathrm{U}(1, 10)$	1
Egg-to-adult development rate, $\gamma(T)$	α	$\Gamma(9, 10^{-5})$	$\mathrm{U}(10^{-5}, 10^{-3})$	5×10^{-6}
	T_0	$\Gamma(7, 2)$	$\mathrm{U}(0, 20)$	0.5
	T_m	$\Gamma(10, 4)$	$\mathrm{U}(20, 50)$	0.5
	σ^2	$\mathrm{U}(0, 1)$	$\mathrm{U}(0, 1)$	0.01

Table S2. Technical details of the MCMC procedure used to determine the temperature-dependent responses of $p(T)$ and $1/g(T)$. Due to the symmetric nature of the data for these parameters, quadratic equations were used (equation S2). The notation $\Gamma(x, y)$ represents a gamma distribution with shape parameter x and scale parameter y, and $\mathrm{U}(x, y)$ is a continuous uniform distribution with bounds x and y. The initial value in the MCMC chain of each sub-parameter was sampled from the distributions listed in the fourth column.

Parameter	Sub-parameter	Prior	Initial value	Σ Value
Egg-to-adult survival probability, $p(T)$	α	$\Gamma(7, 0.001)$	$\mathrm{U}(0.0001, 0.01)$	0.0001
	T_0	$\Gamma(7, 2)$	$\mathrm{U}(0, 20)$	0.5
	T_m	$\Gamma(10, 4)$	$\mathrm{U}(20, 50)$	0.5
	σ^2	$\mathrm{U}(0, 5)$	$\mathrm{U}(0, 2)$	0.01
Adult lifespan, $1/g(T)$	α	$\Gamma(1, 0.5)$	$\mathrm{U}(0.01, 1)$	0.015
	T_0	$\Gamma(5, 2)$	$\mathrm{U}(0, 20)$	1
	T_m	$\Gamma(9, 5)$	$\mathrm{U}(20, 50)$	1
	σ^2	$\mathrm{U}(0, 50)$	$\mathrm{U}(0, 20)$	1

Rainfall-dependent model parameters

Two ecological model parameters were assumed to depend on rainfall: the aquatic stage carrying capacity ($K(R)$) and the rate at which aquatic stage individuals are washed away (the larval flush out rate, $c(R)$). Relationships between the amount of rainfall and the values of these parameters were derived using the approach of Tompkins and Ermert.[4]

Aquatic stage carrying capacity, $K(R)$

The aquatic stage carrying capacity is defined as $K(R) = k(R)D$, in which $k(R)$ is the carrying capacity per unit area and *D* is the area of the location under consideration. We defined $k(R) = w(R)\frac{M_L}{m}$, with $w(R)$ denoting the proportion of land covered with *Ae. aegypti* breeding sites, M_L denoting the total mass of aquatic stage individuals that can be present at a breeding site and m denoting the average mass of an individual in the aquatic phase. Following Tompkins and Ermert,[4]

$$\frac{\mathrm{d}w(R)}{\mathrm{d}t} = \kappa\big[R\big(w_{max} - w(R)\big) - w(R)(\eta + \zeta)\big],$$

in which w_{max} is the proportion of the land surface that is covered by depressions that can become filled with water. The parameter κ is based on the geometry of the depressions and sets the overall rate at which they are filled with or lose water, and the parameters η and ζ determine the relative rates at which water evaporates and is infiltrated into the ground, respectively.

The equilibrium value of $w(R)$ is then

$$w(R) = \frac{Rw_{max}}{\eta + \zeta + R},$$

so that the aquatic stage carrying capacity per unit area is

$$k(R) = \frac{Rw_{max}}{\eta + \zeta + R}\frac{M_L}{m},$$

and

$$K(R) = \frac{Rw_{max}}{\eta + \zeta + R}\frac{M_L D}{m}.$$

We set $\eta = 5$ mm day^{-1},[4] $\zeta = 245$ mm day^{-1},[4] $M_L = 300$ mg m^{-2},[4] and $m = 4.59$ mg (this is the average mass from a sample of 1,000 male and 1,000 female pupae[5]). We set $w_{max} = \frac{1}{25}$, so that 4% of the land's surface can become filled with water.[4] When the rainfall is below 0.2 mm day^{-1}, we set $k(R) = 0$ (and therefore $K(R) = 0$), since standing water is required for vector breeding and development.

In the expression above, when there is a very large amount of rainfall, $k(R)$ (and therefore $K(R)$) tends to a constant value (the maximum possible carrying capacity). The dependence of $k(R)$ on R is shown for values of R between 0 and 25 mm day^{-1} in Fig S6E. For comparison, we also plot the (constant) carrying capacity from a model developed by Silva *et al.*[2] The study location in that research was Nova Iguaçu, Brazil which has an average daily

rainfall of 4.89 mm. The carrying capacity from the model of Silva *et al.*[2] is consistent with the corresponding value of $k(R)$ in our model at that level of rainfall (Fig S6E).

Larval flush out rate, $c(R)$

Each day, the probability that an aquatic stage individual survives being washed away is assumed to have the form[4]

$$K_f(R) = C_1 + C_2 \exp(-C_3 R).$$

We assume that, in the absence of rainfall, individuals will not be washed away, and for large amounts of rainfall, individuals will definitely be washed away. Consequently, $K_f(0) = 1$ and $K_f(R) \to 0$ as $R \to \infty$. Setting $C_3 = 1$, so that the daily survival probability is 0.25 at a moderate rainfall level of 500 mm year^{-1},[6] gives $K_f(R) = \exp(-R)$.

We note that, in the ecological model, larval flush out occurs at (exponential) rate $c(R)$. Hence, the probability of an aquatic stage individual surviving any single day is $\exp(-c(R))$. Matching this with the expression above gives $c(R) = R$ (Fig S6F).

We also conducted supplementary analyses in which we considered the sensitivity of our results to the shape of the ecological niche by considering different assumptions about the relationship between rainfall and the rate at which aquatic stage individuals are washed away. Specifically, we also considered scenarios in which $c(R) = 2R$ (Figs S9A and S10A) and $c(R) = \frac{1}{2}R$ (Figs S9B and S10B). In each case that we considered, our qualitative finding about the likely future poleward spread of *Ae. aegypti* was unchanged.

Supplementary results using ecological niches from other studies

In most of the main text (except for the analysis shown in Fig 4), we used the mechanistic model of *Ae. aegypti* ecological dynamics described above. To show that natural climate

variability is a large driver of uncertainty in future environmental suitability for a range of vector species (and also for specific pathogens), we conducted supplementary analyses using ecological niches derived from other studies in the literature. Relevant results are shown in Fig 4 of the main text, Fig S13 and Table S3.

Table S3. Effect of natural climate variability on environmental suitability for a range of vectors and pathogens, using ecological niches from the literature. The vector (and pathogen, where applicable) under consideration is listed along with the corresponding ecological niche. Environmental suitability in 2100 is then computed for every CESM climate projection in every pixel globally. The results are given in the form (X, Y, Z), where X is the minimum number of months that are projected to be suitable across all climate projections, Y is the corresponding number of months when the mean of the climate projections is used, and Z is the maximum number of months that are projected to be suitable across all climate projections. *For the model of Liu-Helmersson *et al.*[7], we derived the ecological niche by calculating the equilibria for different temperature-rainfall values under the baseline model parameterisation in that article (as in our main analyses, the ecological niche is assumed to represent temperature-rainfall values for which a positive equilibrium vector population size exists).

Vector/pathogen (reference)	Temperature niche (°C)	Rainfall niche (mm day^{-1})	Year	London	Cape Town	Islamabad
Ae. aegypti[1]	(17.8, 34.6)	-	2060<br>2100	(1, 4, 4)<br>(2, 4, 5)	(6, 7, 9)<br>(8, 9, 12)	(5, 7, 8)<br>(5, 6, 9)
Ae. aegypti[8]	(21.3, 34.0)	-	2060<br>2100	(0, 0, 2)<br>(0, 2, 4)	(0, 1, 3)<br>(2, 4, 6)	(3, 6, 7)<br>(4, 5, 6)
Ae. aegypti[7]	*See caption	*See caption	2060<br>2100	(2, 4, 5)<br>(3, 4, 6)	(4, 9, 11)<br>(9, 12, 12)	(6, 7, 10)<br>(6, 9, 11)
Ae. albopictus[1]	(16.2, 31.6)	-	2060<br>2100	(2, 4, 5)<br>(4, 4, 6)	(8, 10, 12)<br>(11, 12, 12)	(4, 5, 7)<br>(5, 7, 9)
Ae. albopictus[8]	(19.9, 29.4)	-	2060<br>2100	(0, 2, 4)<br>(0, 3, 4)	(3, 4, 6)<br>(4, 6, 8)	(2, 5, 5)<br>(2, 4, 6)
An. stephensi/P. falciparum[9]	(16.0, 36.5)	-	2060<br>2100	(2, 4, 5)<br>(4, 4, 6)	(8, 11, 12)<br>(12, 12, 12)	(7, 7, 10)<br>(6, 9, 10)
An. stephensi/P. vivax[9]	(16.6, 31.7)	-	2060<br>2100	(2, 4, 5)<br>(3, 4, 6)	(7, 9, 12)<br>(9, 12, 12)	(4, 5, 7)<br>(5, 7, 9)
Anopheles[10]	(12.16, 40)	(0, 50)	2060<br>2100	(5, 6, 8)<br>(5, 6, 9)	(12, 12, 12)<br>(12, 12, 12)	(11, 12, 12)<br>(10, 11, 12)

Supplementary Figures

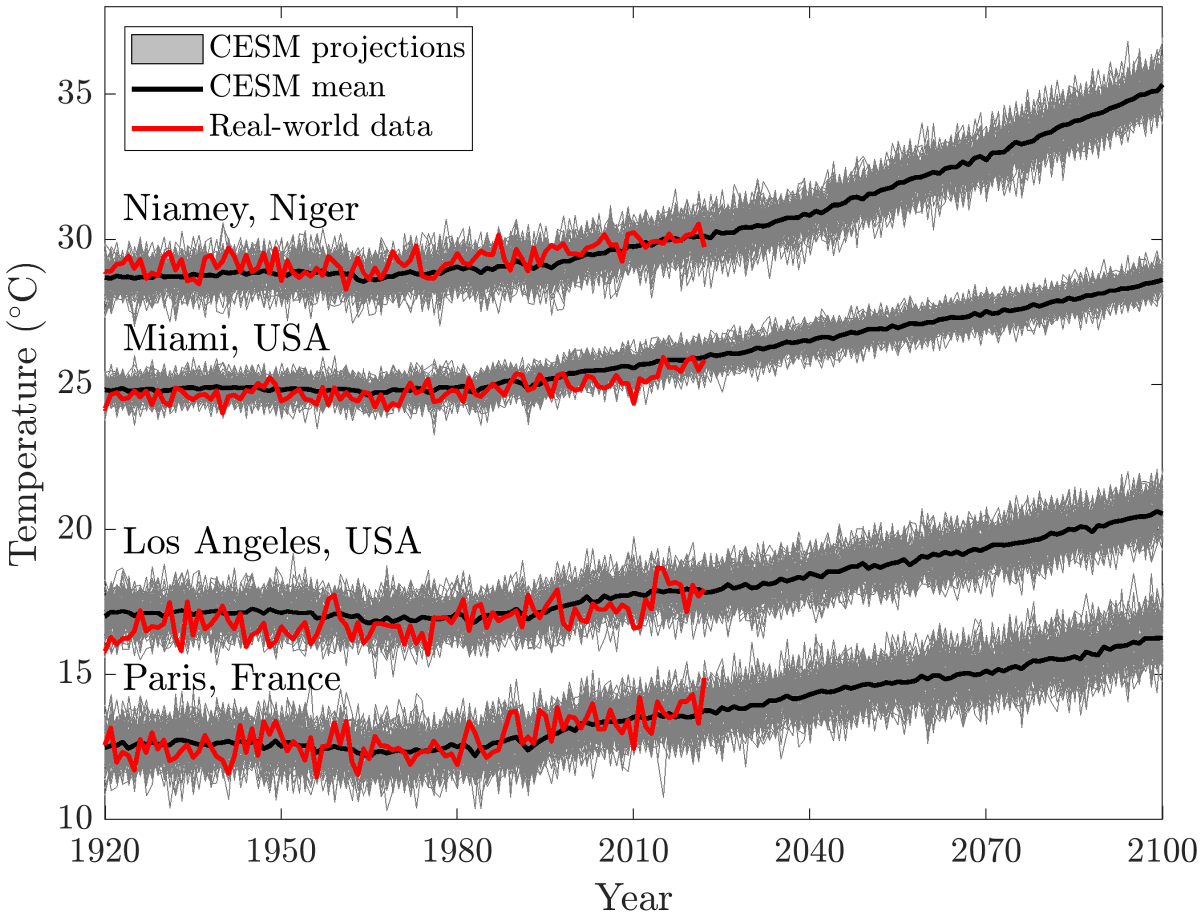

Figure S1. The variability in real-world climate data exceeds that of the CESM ensemble mean. As an example, temperature data were extracted from the CESM LENS2 dataset in the locations of Miami, Los Angeles, Paris and Niamey covering the period from 1920-2100, and the yearly mean was considered. This figure indicates the signal due to anthropogenic climate change (mean of the CESM projections – black), the individual CESM projections (grey) and the temperature observed in the real-world from 1920-2022 (extracted from the Berkeley Earth Surface Temperatures online database;[11] red). This indicates that the variability in real-world climate data is more accurately represented by the variability across the CESM simulations, rather than the ensemble mean.

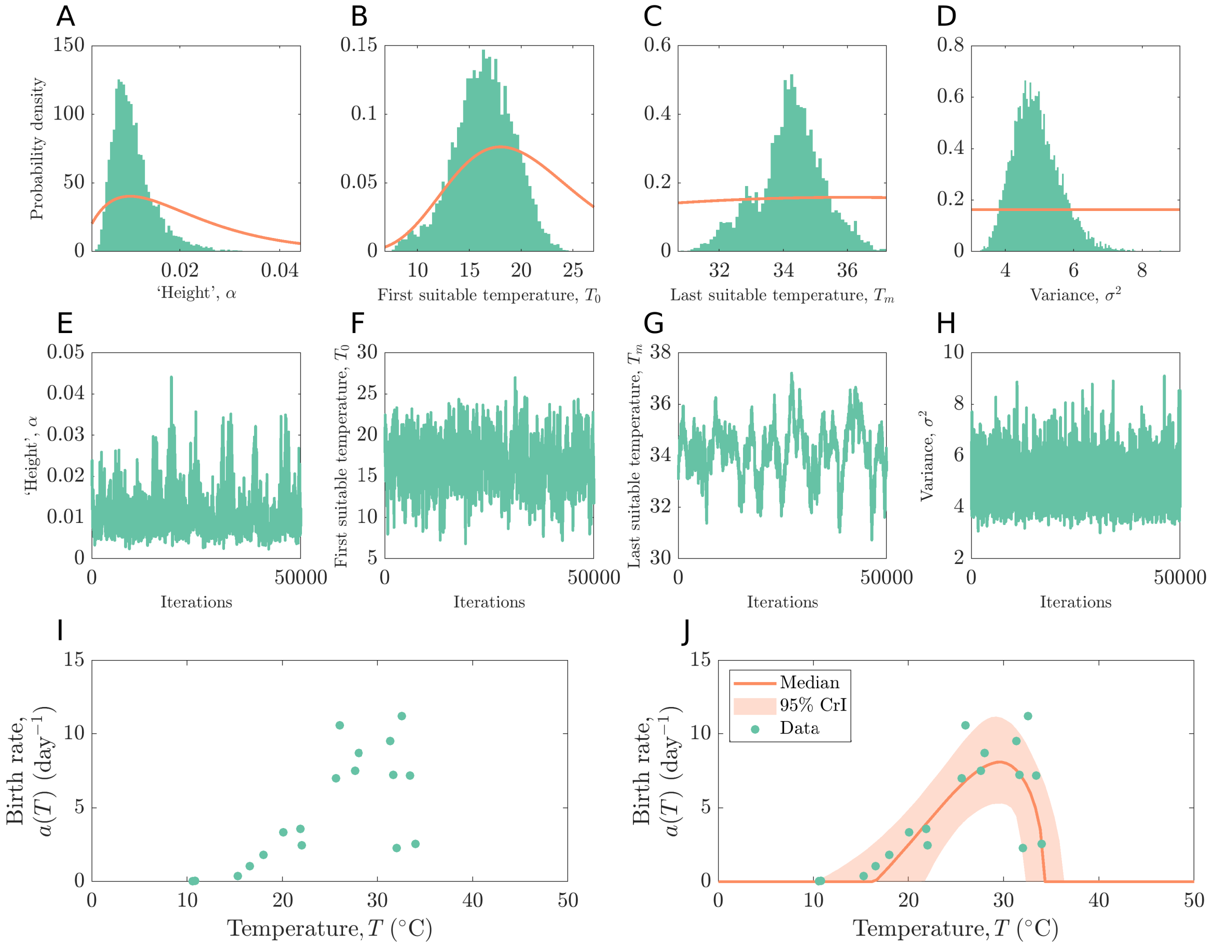

Figure S2. The dependence of the *Ae. aegypti* birth rate ($a(T)$) on temperature. A-D. Prior (orange) and posterior (green) distributions for each of the fitted sub-parameters (α, T_0, T_m and σ^2). To allow the posterior distribution to be seen clearly, x-axes limits are restricted to the minimum and maximum values in the posterior. E-H. Trace plots corresponding to the posterior distributions shown in panels A-D. 100,000 steps were run in the MCMC chain, with the first 50,000 discarded as burn-in (acceptance rate: 0.2419). Five chains were run to compute the Gelman-Rubin statistic (which was 1.0141, 1.0015, 1.0222 and 1.0034 for α, T_0, T_m and σ^2, respectively); the trace plots in panels E-H are from the first chain. I. Data describing the *Ae. aegypti* birth rate as a function of temperature. J. Brière equation fit to the data in panel I (data – green; median fit – orange; 95% equal-tailed credible interval – shaded region).

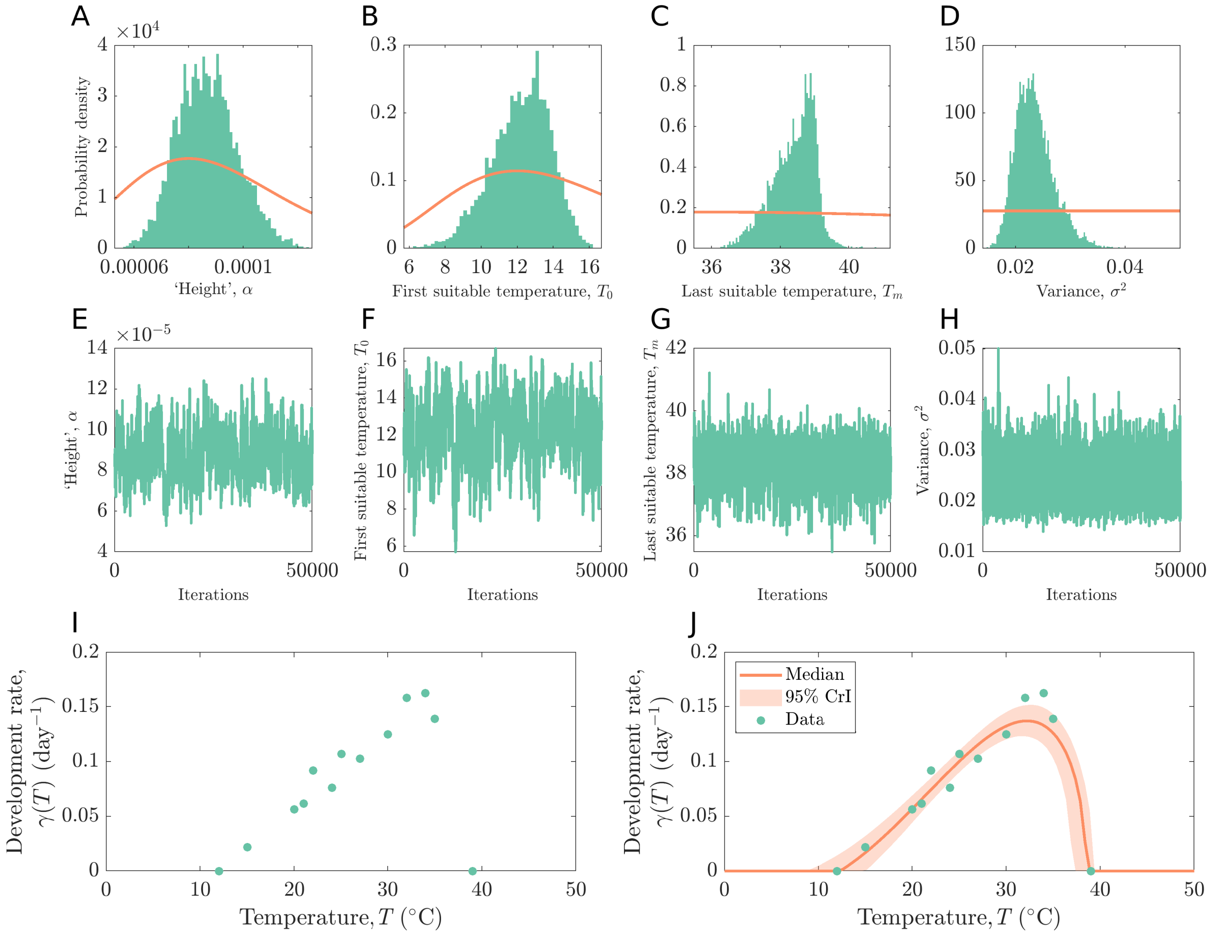

Figure S3. The dependence of the *Ae. aegypti* egg-to-adult development rate ($\gamma(T)$) on temperature. A-D. Prior (orange) and posterior (green) distributions for each of the fitted sub-parameters (α, T_0, T_m and σ^2). To allow the posterior distribution to be seen clearly, x-axes limits are restricted to the minimum and maximum values in the posterior. E-H. Trace plots corresponding to the posterior distributions shown in panels A-D. 100,000 steps were run in the MCMC chain, with the first 50,000 discarded as burn-in (acceptance rate: 0.1982). Five chains were run to compute the Gelman-Rubin statistic (which was 1.0008, 1.0004, 1.0010 and 1.0002 for α, T_0, T_m and σ^2, respectively); the trace plots in panels E-H are from the first chain. I. Data describing the *Ae. aegypti* egg-to-adult development rate as a function of temperature. J. Brière equation fit to the data in panel I (data – green; median fit – orange; 95% equal-tailed credible interval – shaded region).

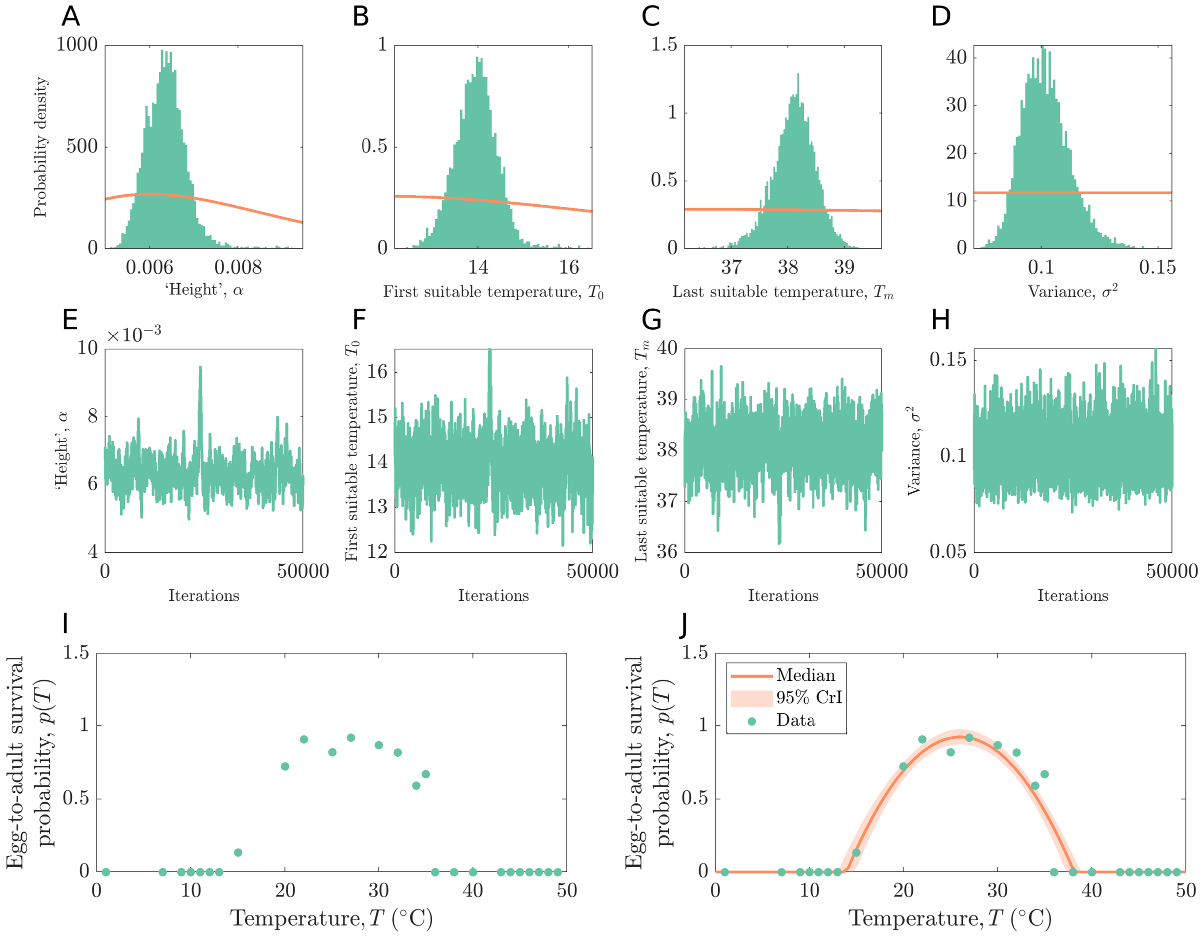

Figure S4. The dependence of the *Ae. aegypti* egg-to-adult survival probability ($p(T)$) on temperature. A-D. Prior (orange) and posterior (green) distributions for each of the fitted sub-parameters (α, T_0, T_m and σ^2). To allow the posterior distribution to be seen clearly, x-axes limits are restricted to the minimum and maximum values in the posterior. E-H. Trace plots corresponding to the posterior distributions shown in panels A-D. 100,000 steps were run in the MCMC chain, with the first 50,000 discarded as burn-in (acceptance rate: 0.2267). Five chains were run to compute the Gelman-Rubin statistic (which was 1.0048, 1.0031, 1.0024 and 1.0004 for α, T_0, T_m and σ^2, respectively); the trace plots in panels E-H are from the first chain. I. Data describing the *Ae. aegypti* egg-to-adult survival probability as a function of temperature. J. Quadratic equation fit to the data in panel I (data – green; median fit – orange; 95% equal-tailed credible interval – shaded region). Fitted values were constrained to lie between zero and one so that $p(T)$ represents a valid probability (MCMC steps corresponding to values outside of this range were discarded).

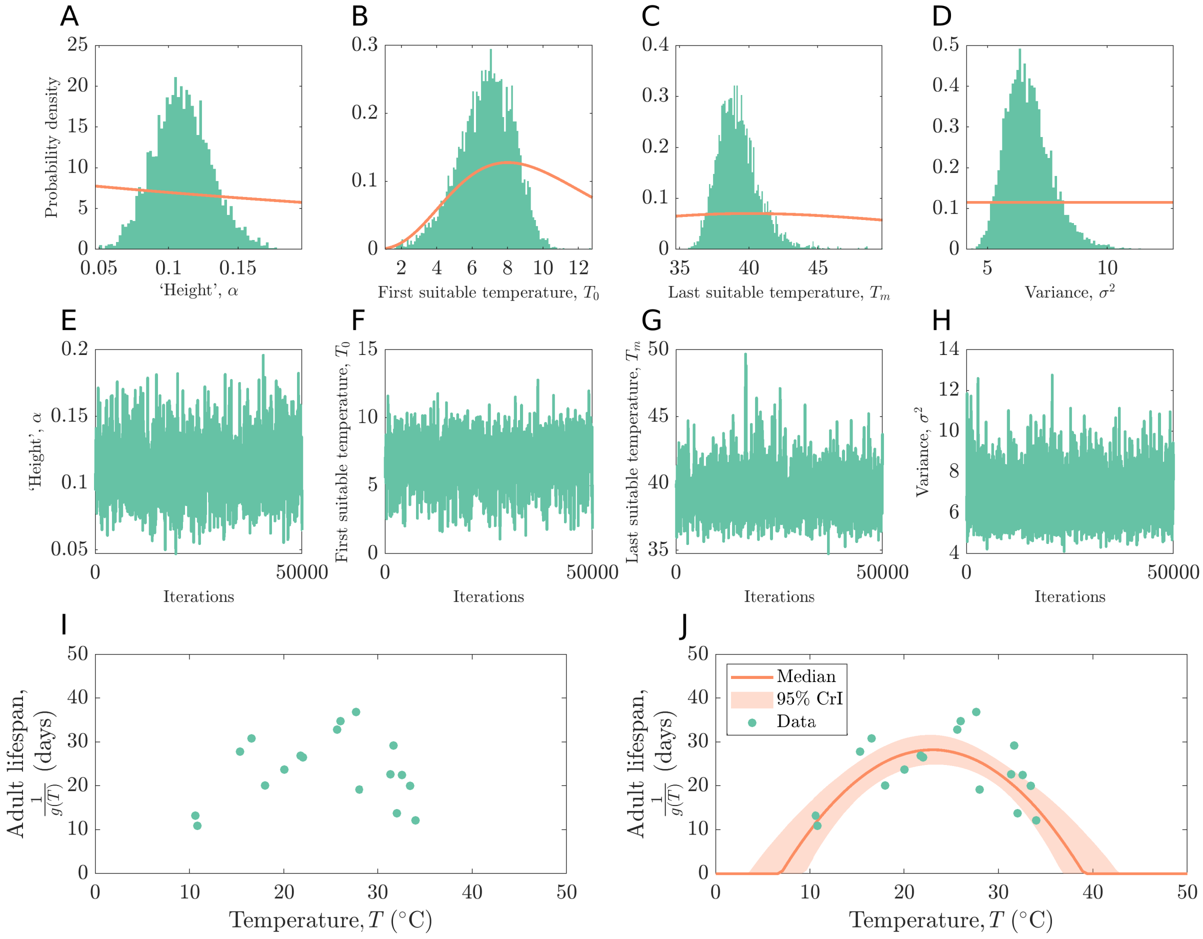

Figure S5. The dependence of the *Ae. aegypti* adult lifespan ($\mathbf{1/g(T)}$) on temperature. A-D. Prior (orange) and posterior (green) distributions for each of the fitted sub-parameters (α, T_0, T_m and σ^2). To allow the posterior distribution to be seen clearly, x-axes limits are restricted to the minimum and maximum values in the posterior. E-H. Trace plots corresponding to the posterior distributions shown in panels A-D. 100,000 steps were run in the MCMC chain, with the first 50,000 discarded as burn-in (acceptance rate: 0.2141). Five chains were run to compute the Gelman-Rubin statistic (which was 1.0008, 1.0003, 1.0014 and 1.0001 for α, T_0, T_m and σ^2, respectively); the trace plots in panels E-H are from the first chain. I. Data describing the *Ae. aegypti* adult lifespan as a function of temperature. J. Quadratic equation fit to the data in panel I (data – green; median fit – orange; 95% equal-tailed credible interval – shaded region).

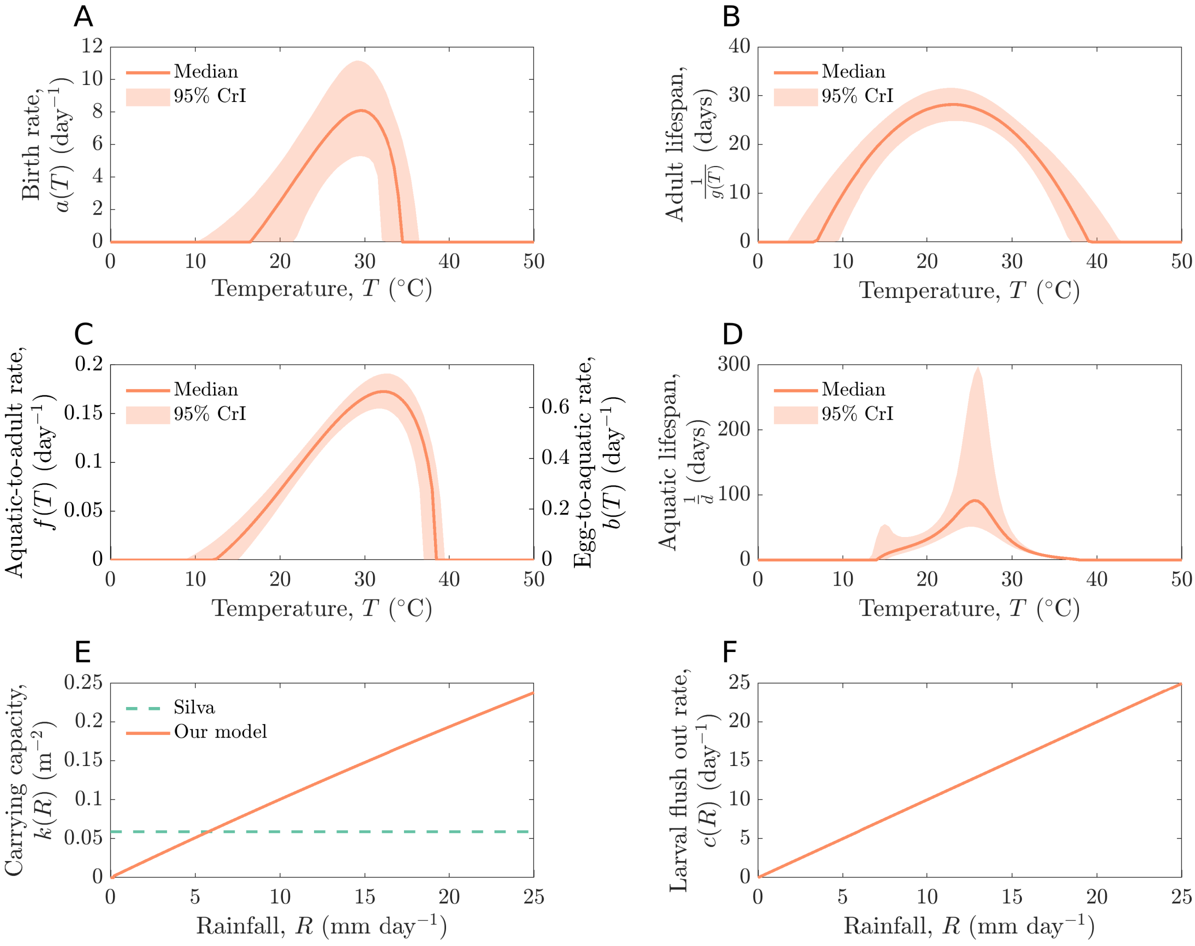

Figure S6. The dependence of the parameters of the ecological model on temperature and rainfall. Posterior distributions are shown for temperature-dependent parameters (median fit – orange; 95% equal-tailed credible interval – shaded region): A. Birth rate ($a(T)$); B. Adult lifespan ($1/g(T)$); C. Aquatic-to-adult development rate (left y-axis, $f(T)$) and egg-to-aquatic development rate (right y-axis, $b(T)$); D. Aquatic stage lifespan ($1/d(T)$). Rainfall-dependent parameter responses (orange) for: E. Aquatic stage carrying capacity per unit area ($k(R)$); F. Larval flush out rate ($c(R)$). In panel E, the aquatic stage carrying capacity estimated in a previous study[2] is also plotted for comparison (green dotted); in that study location (Nova Iguaçu, Brazil), the average rainfall is 4.89 mm per day.

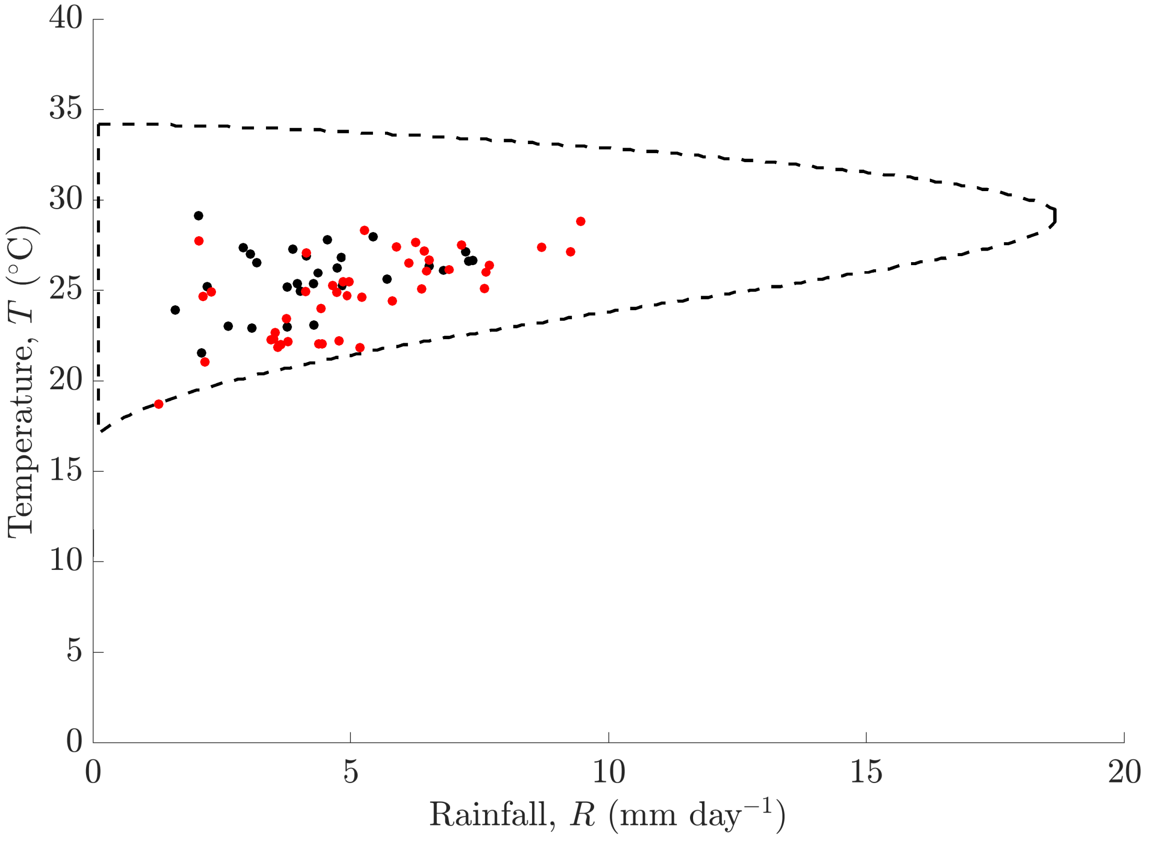

Figure S7. Comparison of the ecological niche derived from our model and real-world data. The black dotted line is the 50th percentile ecological niche (Fig 1C in the main text). Black dots indicate the mean temperature and rainfall in 29 countries with confirmed *Ae. aegypti* populations (locations were reported in Kraemer *et al.*[12]; temperature and rainfall values were extracted from the World Bank's Climate Change Knowledge Portal[13] and represent averaged values across the year 2015). Red dots indicate temperature and rainfall values for 48 locations that have experienced outbreaks of dengue virus disease (locations were reported by Liu *et al.*[14], and temperature and rainfall values for each outbreak are mean values across the period of the outbreak extracted from the World Bank's Climate Change Knowledge Portal[13] and the National Centers for Environmental Information's Climate Data Online tool[15]).

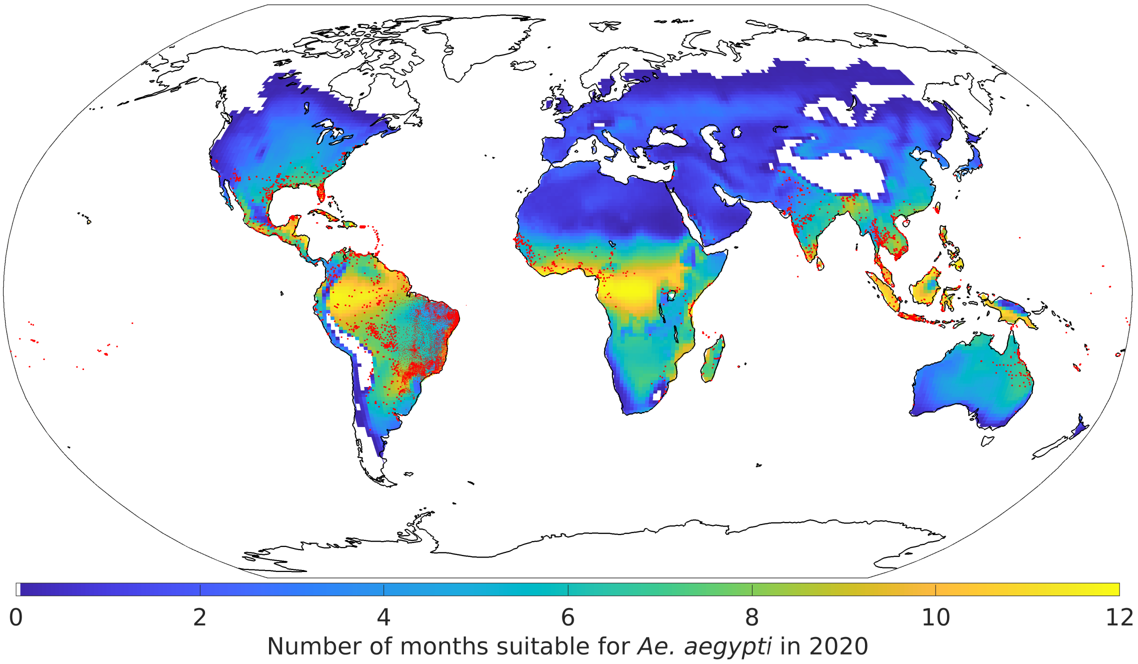

Figure S8. Comparison of model-predicted global suitability for *Ae. aegypti* in 2020 and known locations with *Ae. aegypti* prior to and including 2020. The number of months that are predicted to be suitable for *Ae. aegypti* in different locations globally in 2020. These results were obtained first for each CESM simulation individually, and then averaged across all CESM simulations. Red dots indicate locations in which *Ae. aegypti* have been reported, as described in a previous research article.[12]

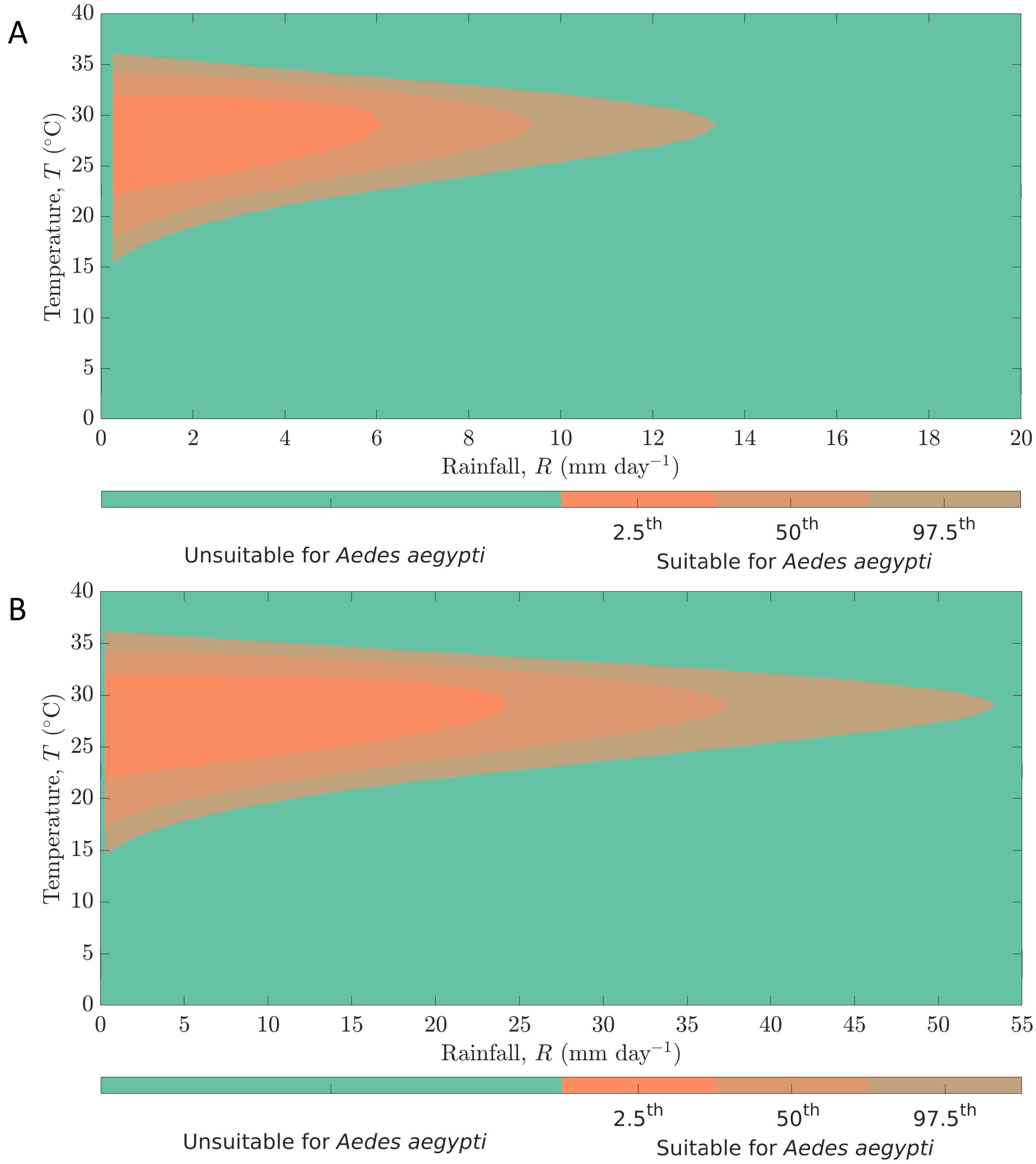

Figure S9. Sensitivity of the ecological niche to the relationship between rainfall and the probability that aquatic stage individuals are washed away. A. The ecological niche derived from the ecological model when aquatic stage individuals are washed away at a faster rate than assumed in our main analyses ($c(R) = 2R$). B. The ecological niche derived from the ecological model when aquatic stage individuals are washed away at a slower rate than assumed in our main analyses ($c(R) = \frac{1}{2}R$). Uncertainty in the ecological niche is represented by different shades of orange and arises due to uncertainty in the parameter estimates of the ecological model.

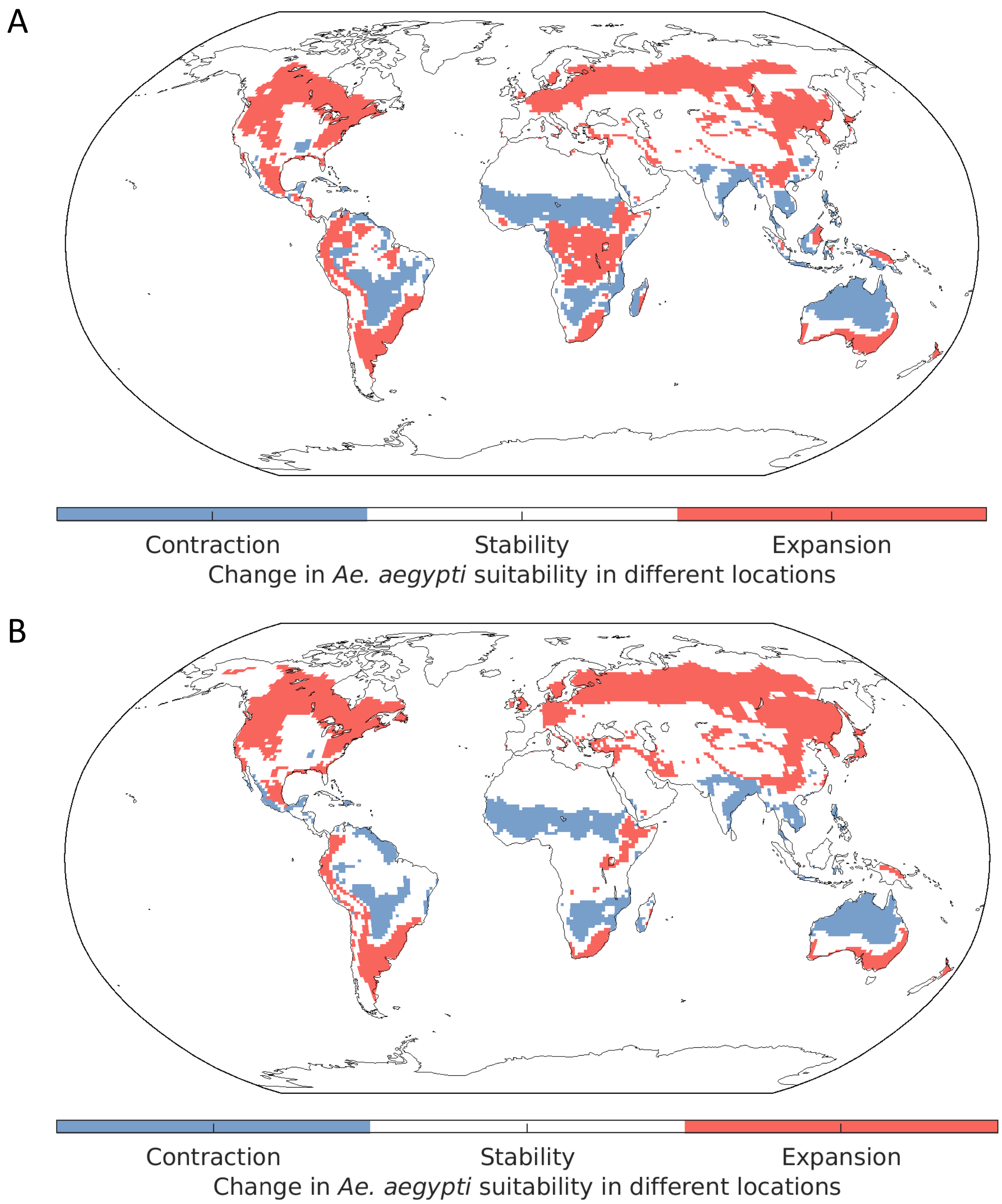

Figure S10. Locations that are expected to see an increase or decrease in the suitability of climatic conditions for *Ae. aegypti*, for different ecological niches. Results shown here are analogous to those in Fig 2 in the main text, but for: A. The ecological niche shown in Fig S9A ($c(R) = 2R$). B. The ecological niche shown in Fig S9B ($c(R) = \frac{1}{2}R$). Locations in which the number of months that are suitable for *Ae. aegypti* increases by at least one in 2100 compared to 2020 are shown in red. Locations with a corresponding decrease are shown in blue. In each panel, the results were obtained by first calculating the change in the number of suitable months for each CESM projection individually, and then averaging across all projections.

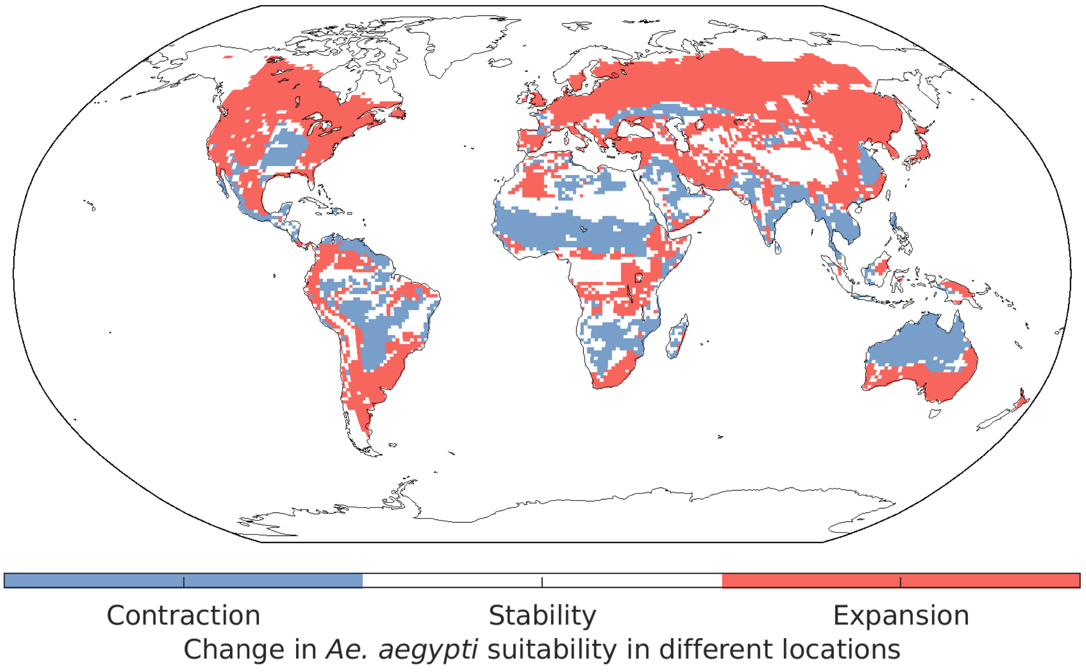

Figure S11. Locations that are expected to see an increase or decrease in the suitability of climatic conditions for *Ae. aegypti*, based on the mean of the CESM climate projections. Results shown here are analogous to those in Fig 2 in the main text, but instead averaging the climate data (across all CESM simulations) prior to calculating the number of months of suitable conditions for *Ae. aegypti* in 2020 and 2100. Locations in which the number of months that are suitable for *Ae. aegypti* increases by at least one in 2100 compared to 2020 are shown in red. Locations with a corresponding decrease are shown in blue.

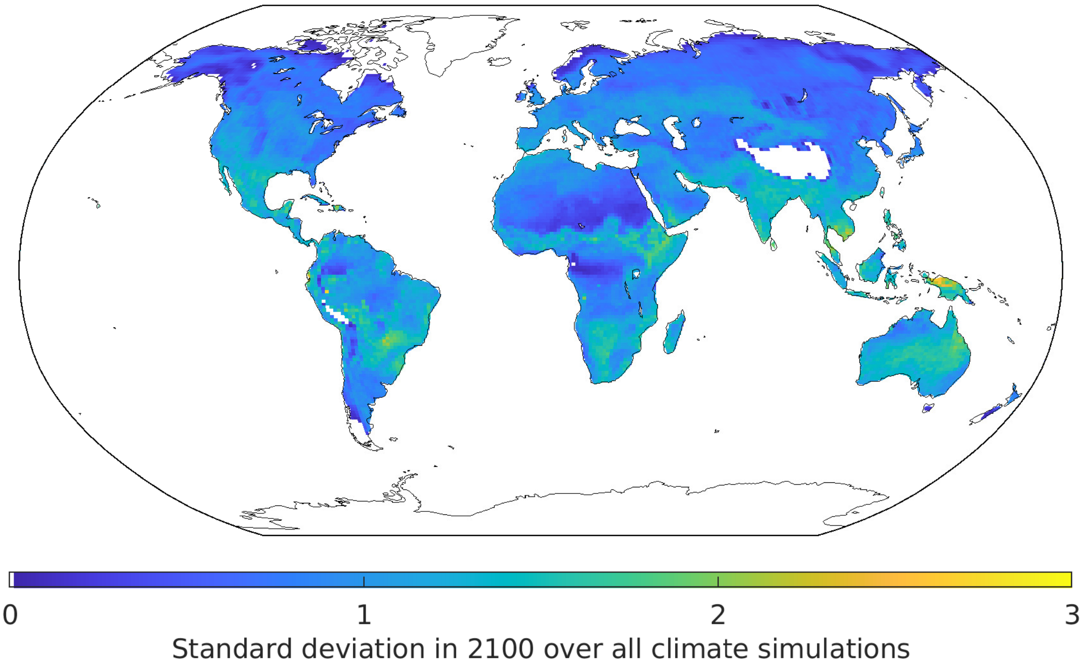

Figure S12. Geographical variability in the impact of natural climate variability on climate suitability for *Ae. aegypti* in different locations. The standard deviation in the number of months that are projected to be suitable for *Ae. aegypti* in the year 2100 across the CESM projections. Values are computed from the full range of 100 CESM projections.

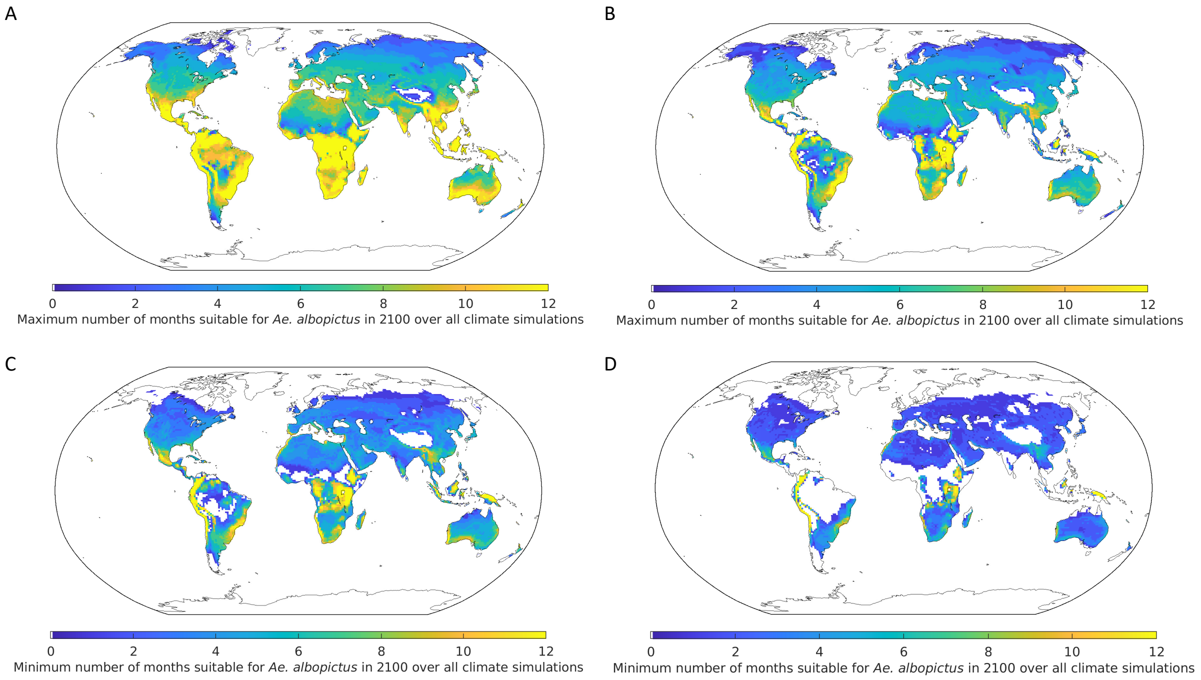

Figure S13. The impact of natural climate variability on future suitability for *Ae. albopictus* in different locations using ecological niches from the literature. A. The maximum number of months that are projected to be suitable for *Ae. albopictus* in the year 2100 using the ecological niche from Mordecai *et al.*[1] B. Identical to panel A, but using the ecological niche from Ryan *et al.*[8] C. The minimum number of months that are projected to be suitable for *Ae. albopictus* in the year 2100 using the ecological niche from Mordecai *et al.*[1] D. Identical to panel C, but using the ecological niche from Ryan *et al.*[8] In this figure, for each latitude-longitude value, the CESM projection corresponding to the most (panels A-B) or fewest (panel C-D) number of months that are suitable for *Ae. albopictus* in the year 2100 is chosen.

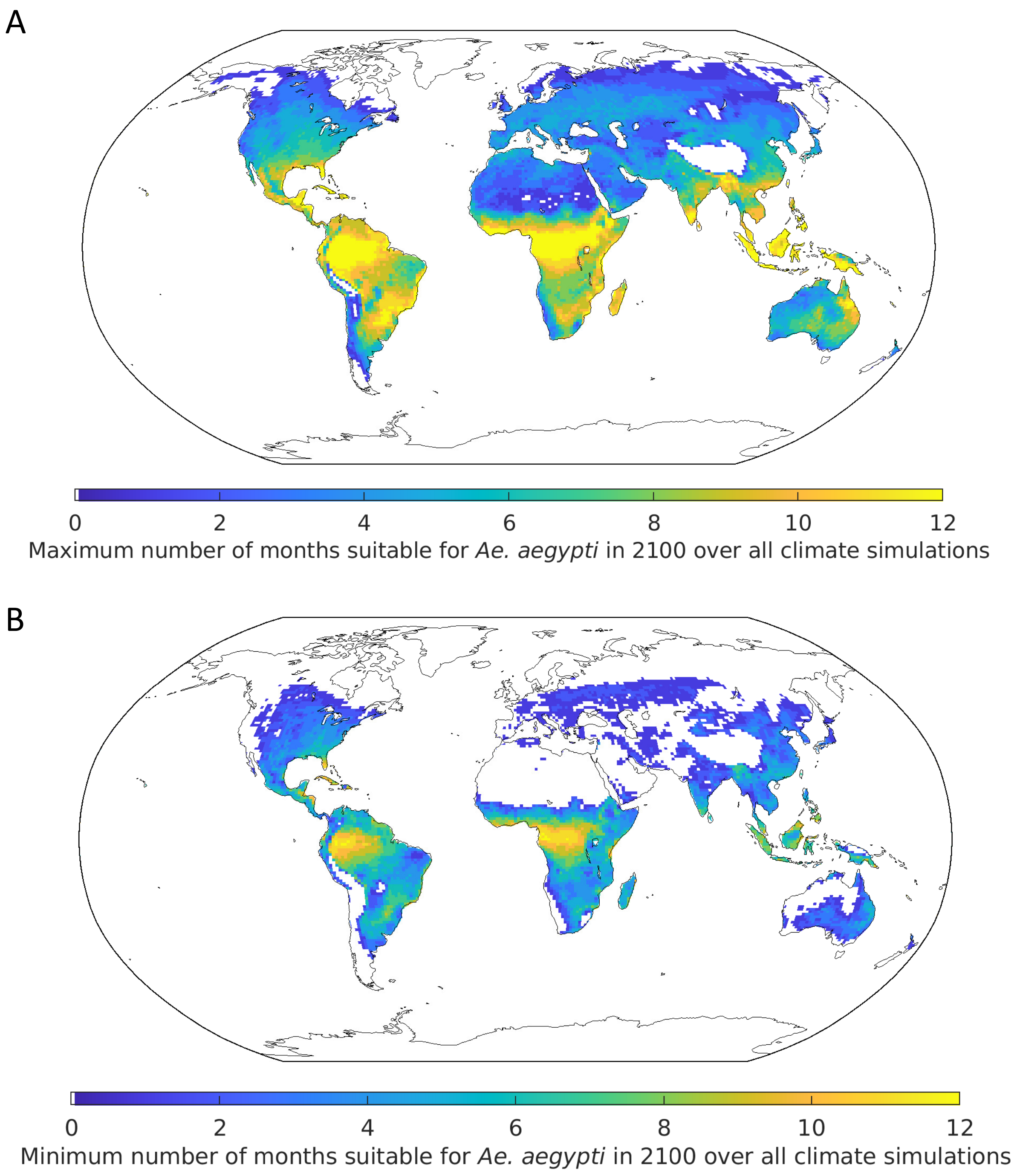

Figure S14. **The impact of natural climate variability on future environmental suitability for *Ae. aegypti* in different locations under SSP2-4.5.** A. The maximum number of months that are projected to be suitable for *Ae. aegypti* in the year 2100. B. The minimum number of months that are projected to be suitable for *Ae. aegypti* in the year 2100. In both panels, for each latitude-longitude value, the CESM projection corresponding to the most (panel A) or fewest (panel B) number of months that are suitable for *Ae. aegypti* in the year 2100 is chosen. These results were constructed using 19 projections from the CESM under SSP2-4.5.

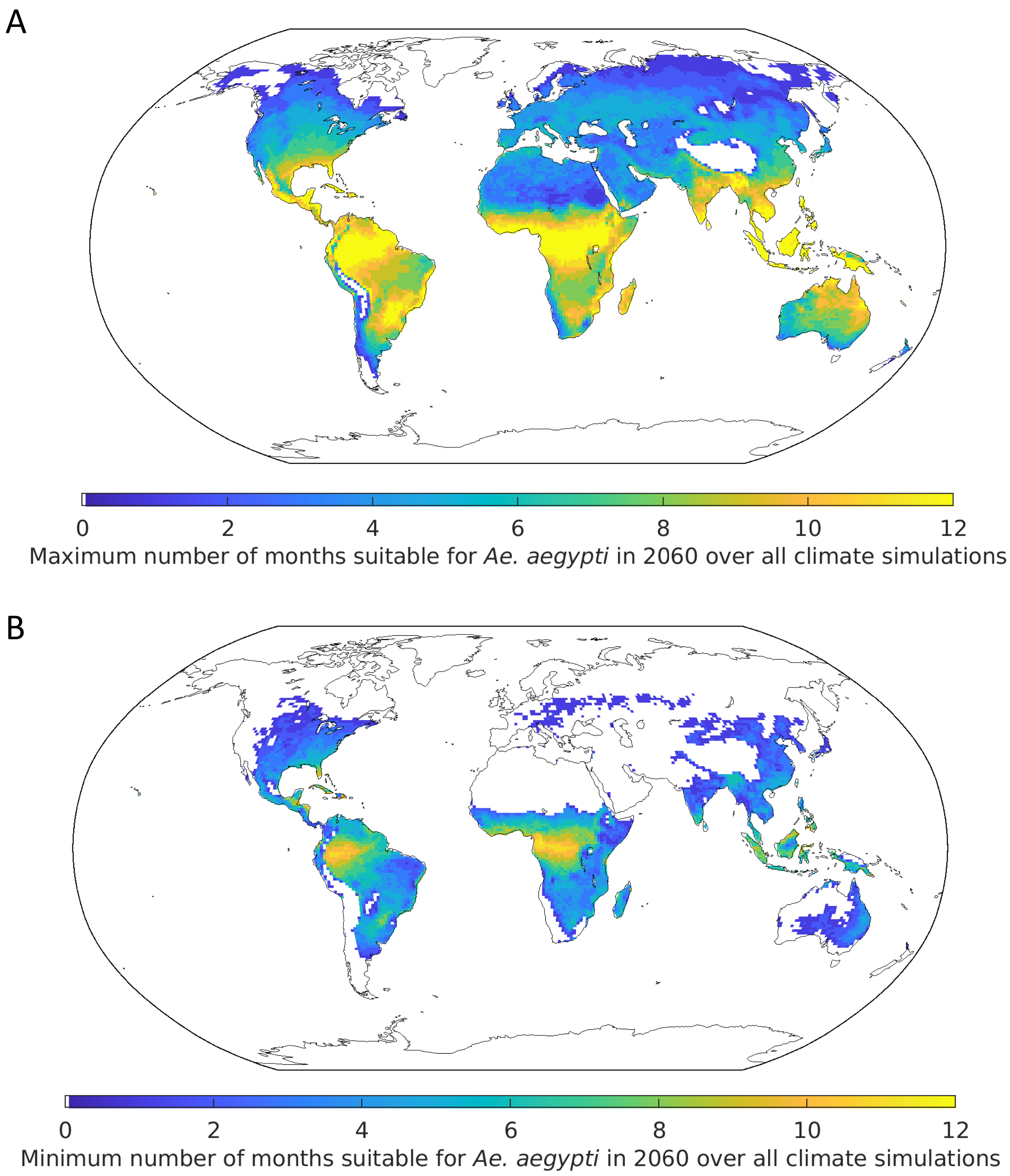

Figure S15. The impact of natural climate variability on projected suitability for *Ae. aegypti* in 2060 in different locations. A. The maximum number of months that are projected to be suitable for *Ae. aegypti* in the year 2060. B. The minimum number of months that are projected to be suitable for *Ae. aegypti* in the year 2060. In both panels, for each latitude-longitude value, the CESM projection corresponding to the most (panel A) or fewest (panel B) number of months that are suitable for *Ae. aegypti* in the year 2060 is chosen. This figure is analogous to Fig 3A-B in the main text, but for the year 2060 rather than 2100.

A
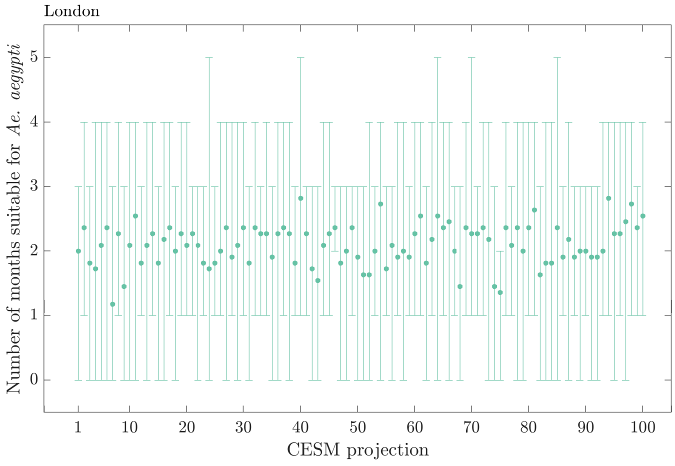

B
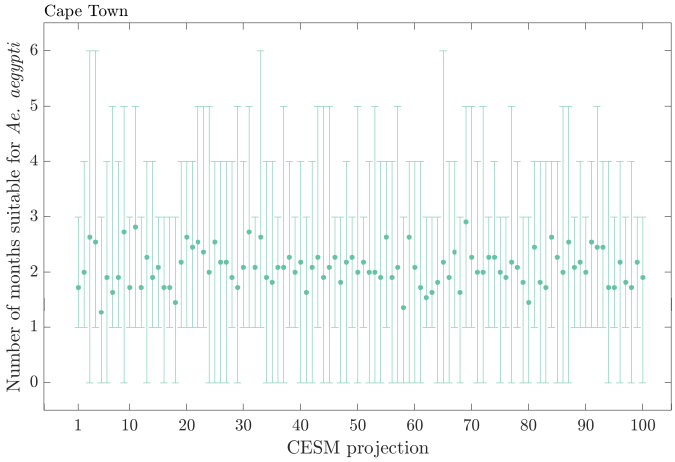

C
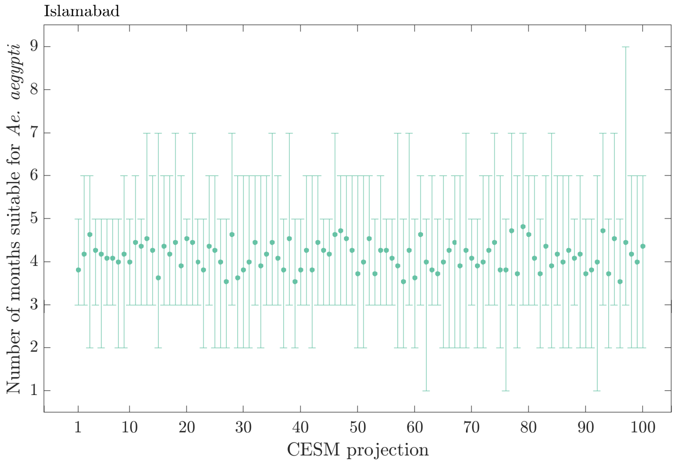

Figure S16. Variability in environmental suitability for *Ae. aegypti* between years in the period from 2090-2100. A. The number of months that are projected to be suitable for *Ae. aegypti* each year in London for each climate simulation (labelled from 1-100). Dots represent mean values (across all years in the period 2090-2100), with whiskers showing the range between years. B. Analogous to panel A, but for Cape Town. C. Analogous to panel A, but for Islamabad.

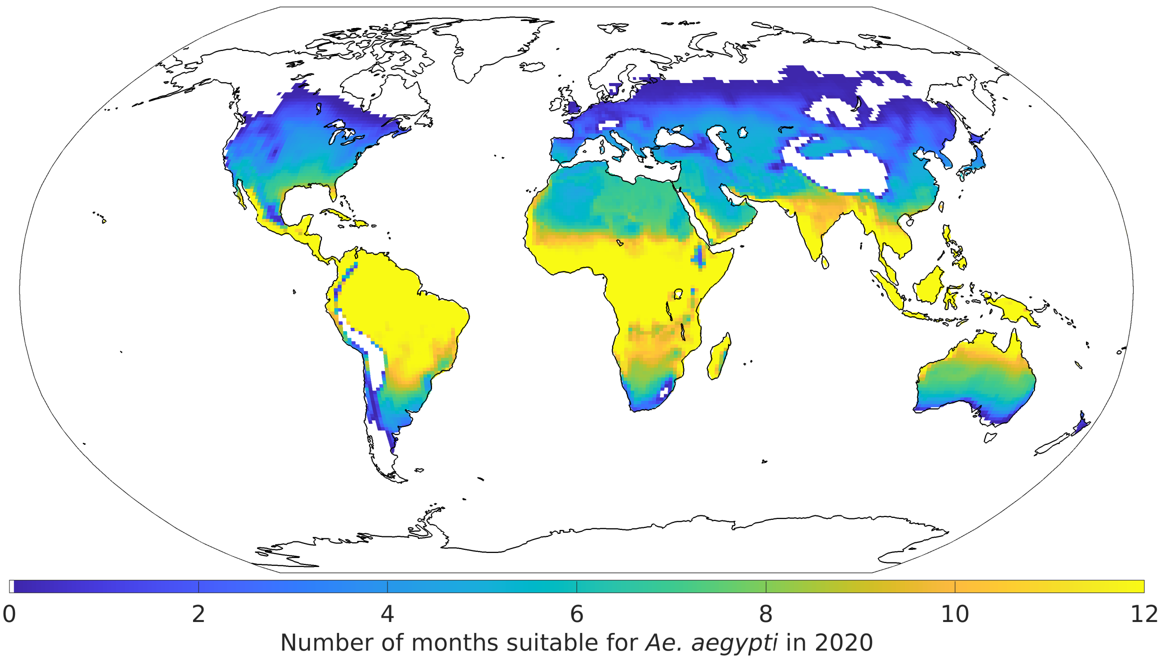

Figure S17. Model-predicted global suitability for *Ae. aegypti* in 2020 if rainfall is assumed not to vary spatially. The number of months that are predicted to be suitable for *Ae. aegypti* in different locations globally in 2020, if rainfall is fixed at 3 mm day^{-1} everywhere. These results were obtained for each CESM simulation individually, before averaging the number of months of environmental suitability across the CESM simulations. By comparing these results with those shown in Fig S8, the effect of rainfall in reducing the range of locations with suitable conditions for *Ae. aegypti* can be seen.

References

1 Mordecai EA, Cohen JM, Evans MV, *et al.* Detecting the impact of temperature on transmission of Zika, dengue, and chikungunya using mechanistic models. *PLoS Negl Trop Dis* 2017; **27**: e0005568.

2 Silva MR, Lugão PHG, Chapiro G. Modeling and simulation of the spatial population dynamics of the *Aedes aegypti* mosquito with an insecticide application. *Parasites Vectors* 2020; **13**: 550.

3 Roberts GO, Rosenthal JS. Optimal scaling for various Metropolis-Hastings algorithms. *Statist Sci* 2001; **16**: 351–67.

4 Tompkins AM, Ermert V. A regional-scale, high resolution dynamical malaria model that accounts for population density, climate and surface hydrology. *Malar J* 2013; **12**: 65.

5 Kittayapong P, Kaeothaisong N, Ninphanomchai S, Limohpasmanee W. Combined sterile insect technique and incompatible insect technique: sex separation and quality of sterile *Aedes aegypti* male mosquitoes released in a pilot population suppression trial in Thailand. *Parasites Vectors* 2018; **11**: 657.

6 Parham PE, Michael E. Modeling the effects of weather and climate change on malaria transmission. *Environ Health Perspect* 2010; **118**: 620–6.

7 Liu-Helmersson J, Brännström Å, Sewe MO, Semenza JC, Rocklöv J. Estimating past, present, and future trends in the global distribution and abundance of the arbovirus vector *Aedes aegypti* under climate change scenarios. *Front Public Health* 2019; **7**: 148.

8 Ryan SJ, Carlson CJ, Mordecai EA, Johnson LR. Global expansion and redistribution of Aedes-borne virus transmission risk with climate change. *PLoS Negl Trop Dis* 2018; **13**: e0007213.

9 Villena OC, Ryan SJ, Murdock CC, Johnson LR. Temperature impacts the environmental suitability for malaria transmission by *Anopheles gambiae* and *Anopheles stephensi*. *Ecology* 2022; **103**: e3685.

10 Parham PE, Michael E. Modelling climate change and malaria transmission. In: Modelling Parasite Transmission and Control. New York, NY: Springer New York, 2010: 184–99.

11 National Center for Atmospheric Research. The Climate Data Guide: Global surface temperatures (BEST: Berkeley Earth Surface Temperatures). 2023 www.climatedataguide.ucar.edu/climate-data/global-surface-temperatures-best-berkeley-earth-surface-temperatures.

12 Kraemer MUG, Sinka ME, Duda KA, *et al.* The global distribution of the arbovirus vectors *Aedes aegypti* and *Ae. albopictus*. *eLife* 2015; **4**: e08347.

13 World Bank Group. Climate Change Knowledge Portal (CCKP). 2022 https://climateknowledgeportal.worldbank.org/.

14 Liu Y, Lillepold K, Semenza JC, Tozan Y, Quam MBM, Rocklöv J. Reviewing estimates of the basic reproduction number for dengue, Zika and chikungunya across global climate zones. *Environment Res* 2020; **182**: 109114.

15 National Oceanic and Atmospheric Administration. National Centers for Environmental Information: Climate Data Online. 2023. www.ncdc.noaa.gov/cdo-web/.
